# Graphic Warning Labels Elicit Affective and Thoughtful Responses from Smokers: Results of a Randomized Clinical Trial

**DOI:** 10.1371/journal.pone.0142879

**Published:** 2015-12-16

**Authors:** Abigail T. Evans, Ellen Peters, Andrew A. Strasser, Lydia F. Emery, Kaitlin M. Sheerin, Daniel Romer

**Affiliations:** 1 Department of Psychology, Ohio State University, Columbus, Ohio, United States of America; 2 Perelman School of Medicine, University of Pennsylvania, Philadelphia, Pennsylvania, United States of America; 3 Department of Psychology, Northwestern University, Evanston, Illinois, United States of America; 4 Department of Psychological Sciences, University of Missouri, Columbia, Missouri, United States of America; 5 Annenberg Public Policy Center, University of Pennsylvania, Philadelphia, Pennsylvania, United States of America; Aichi Cancer Center Research Institute, JAPAN

## Abstract

**Objective:**

Observational research suggests that placing graphic images on cigarette warning labels can reduce smoking rates, but field studies lack experimental control. Our primary objective was to determine the psychological processes set in motion by naturalistic exposure to graphic vs. text-only warnings in a randomized clinical trial involving exposure to modified cigarette packs over a 4-week period. Theories of graphic-warning impact were tested by examining affect toward smoking, credibility of warning information, risk perceptions, quit intentions, warning label memory, and smoking risk knowledge.

**Methods:**

Adults who smoked between 5 and 40 cigarettes daily (N = 293; mean age = 33.7), did not have a contra-indicated medical condition, and did not intend to quit were recruited from Philadelphia, PA and Columbus, OH. Smokers were randomly assigned to receive their own brand of cigarettes for four weeks in one of three warning conditions: text only, graphic images plus text, or graphic images with elaborated text.

**Results:**

Data from 244 participants who completed the trial were analyzed in structural-equation models. The presence of graphic images (compared to text-only) caused more negative affect toward smoking, a process that indirectly influenced risk perceptions and quit intentions (e.g., image->negative affect->risk perception->quit intention). Negative affect from graphic images also enhanced warning credibility including through increased scrutiny of the warnings, a process that also indirectly affected risk perceptions and quit intentions (e.g., image->negative affect->risk scrutiny->warning credibility->risk perception->quit intention). Unexpectedly, elaborated text reduced warning credibility. Finally, graphic warnings increased warning-information recall and indirectly increased smoking-risk knowledge at the end of the trial and one month later.

**Conclusions:**

In the first naturalistic clinical trial conducted, graphic warning labels are more effective than text-only warnings in encouraging smokers to consider quitting and in educating them about smoking’s risks. Negative affective reactions to smoking, thinking about risks, and perceptions of credibility are mediators of their impact.

**Trial Registration:**

Clinicaltrials.gov NCT01782053

## Introduction

Cigarette smoking causes about 480,000 deaths in the United States (US) each year [1]. In attempts to reduce smoking-related mortality, graphic warning labels on cigarette packaging are now required in 77 countries [2] and were mandated in the US in 2009. However, the DC Circuit Court, a US judicial body that has the authority to invalidate laws it deems unconstitutional, blocked the graphic warning requirement, concluding in part that the images proposed by the Food and Drug Administration (FDA) were unconstitutional and “unabashed attempts to evoke emotion…and browbeat consumers into quitting” [3]. An earlier 2011 memorandum predicted this later ruling: “the emotional response they were crafted to induce is … an objective wholly apart from disseminating purely factual and uncontroversial information” [4]. The aim of the present paper is to examine this statement in light of psychological theory about the effects of emotion on risk perception and informed decision making (from here on, we use the terms emotion, feelings, and affect interchangeably). In particular, we consider the negative affect elicited by graphic warnings to be an important source of health information and driver of cognitive processes critical to informed decisions among smokers. This paper presents data from a randomized clinical trial that is consistent with this notion.

Experimental research aligns with the court’s assertion that graphic warning labels evoke more negative emotion than text-only labels; these negative feelings also generalize to smoking-related cues (e.g., images of cigarettes) [5, 6, 7]. Despite the Court’s assertion, such emotional reactions to danger have been shown critical to the quick perception and reaction to risk that has been necessary for avoiding health and other hazards across human evolutionary history [8]. These emotions can influence behavior directly, motivating us to engage in pleasant, rewarding behaviors and avoid unpleasant, potentially dangerous activities [8]. Affect also acts as an important source of information, informing us whether the water is safe to drink or it’s okay to enter a dark alley (via an Affect Heuristic). In other words, affective responses can serve as simple cues or heuristics enabling people to skirt hazards quickly and efficiently [8, 9]. These are important ways that graphic warning labels may assist smokers, most of whom want to quit and/or wish they had never started smoking [10].

Affect, thus, serves multiple functions in risk perception and decision processes [11, 12]. This line of research also points towards affect influencing perceptions of health risks by motivating people to think more carefully about the risks [11]. Peters [12], for example, proposed that affect plays a role as a spotlight in a two-step process. First, enhanced affective feelings (e.g., those aroused by graphic warnings in the present study) encourage the decision maker to focus on new information consistent with those feelings (in this case, the risk information). Second, the new information (rather than the initial feelings) is used to guide further judgments and decisions. Thus, we expect affect-laden graphic warnings to highlight health-risk information, promote greater processing and acceptance of it, and, in turn, increase smoking risk perceptions and quit considerations. Consistent with this, graphic warnings have been shown to elicit increased evaluations of warning credibility that may encourage quitting [13]. Such cognitions are important because evaluations formed from carefully thinking about information last even longer [14] and are even more predictive of behavior [14, 15, 16]. Understanding the processes through which affect influences risk perceptions and quit intentions among smokers represents the primary objective of the current research.

We further predicted that smokers given graphic warnings would remember more health information from them than those given text-only warnings. Graphic images contain more detailed information than is available in brief textual warnings. For example, showing an image of advanced oral cancer on the lip of a person who also has diseased teeth and gums says more to the smoker than simply saying “cigarettes cause cancer.” In addition, our hypothesis has a basis in past research showing that affective arousal increases memory for associated information [17, 18, 19]. Consistent with both lines of thought, survey research shows that smokers in countries with graphic warning labels can identify more smoking risks than smokers in countries with text-only warnings [20, 21]. Improving our understanding of how graphic warning labels influence smokers’ risk knowledge represents an important secondary objective of this trial.

In addition to images, another label component that can influence the perceived credibility of warnings is the addition of text that elaborates on the risk information in the warnings. Emery et al. [13] found that elaborated risk information (similar to that used in Canada) increased perceived warning credibility, which decreased smoking desires. Because smokers do not appreciate the cumulative risks associated with smoking [22], we designed elaborative text that emphasized the additional risk that each cigarette posed to the smoker. Whether messages emphasizing cumulative risks would be compelling to regular smokers (and especially smokers not intending to quit) was not clear. We anticipated that, consistent with past research showing the superiority of elaborated text [13], such messages would enhance warning credibility, an effect that would indirectly influence risk perceptions and quit intentions.

A recent meta-analysis of 37 laboratory-based experimental studies concluded that one-time exposure to graphic vs. text-only warning labels leads to heightened quit intentions among smokers [23]. However, a naturalistic experiment is a key addition to this literature because, compared to single-exposure studies, the experience of a pack-a-day smoker is likely much different, with 600 potential exposures to graphic warnings on cigarette packs per month. It seems unlikely that the first label response would be the same as the 599^th^. In particular, the effects of graphic images on negative affect towards smoking may be short-lived as smokers could avoid the warnings or habituate to them over time [24, 25]. If true, graphic warnings will lose efficacy over time. However, we anticipated that their emotional impact would change smokers’ feelings about their habit and motivate greater processing of risk information [11], leaving the smoker with enhanced knowledge and heightened risk perceptions even after the immediate emotions waned. The present study, to our knowledge, is the first to randomly assign smokers to receive graphic vs. text-only warnings on their cigarette packs over a relatively long duration (four weeks).

We conducted a randomized clinical trial to experimentally study smokers in a naturalistic setting in order to test causal claims based on longer term exposures than past experiments. We compared three conditions: text-only using the nine Congressionally-mandated messages; mandated text plus FDA-proposed images [26]; and elaborated text combined with the mandated messages and proposed images. Consistent with past research [6, 13, 27], we tested our hypotheses using mediated models with affect, risk scrutiny, perceived credibility, and label memory as mediators of warning label effects on risk perception, quit intentions, and risk knowledge. We tested the following hypotheses:

Hypothesis 1a (H1a): Graphic warning labels will cause greater negative affect toward smoking among adult smokers, an effect that will indirectly motivate action by increasing quit intentions and serve as information about the dangers of smoking by increasing risk perceptions.

Hypothesis 1b (H1b): The negative affect created by graphic images will enhance the credibility of the warnings by increasing scrutiny of the warning information, an effect that will indirectly increase risk perceptions and quit intentions.

Hypothesis 2 (H2): Elaborative text will increase the credibility of the warning message, an effect that will also indirectly increase risk perceptions and quit intentions.

Hypothesis 3 (H3). Graphic warning labels will enhance recall of warning information, an effect that will be explained in part by negative affect elicited by the graphic labels. This greater label memory will indirectly increase participants’ knowledge of smoking risks at the end of the trial and at post-trial follow-up.

## Materials and Methods

The protocol for this trial and CONSORT checklist are available as supplemental information; see S1 Protocol and S1 Checklist. The trial was registered at Clinicaltrials.gov with the reference NCT01782053.

### Ethics statement

This study was conducted in accordance with the ethical standards laid down in the 1964 Declaration of Helsinki. The investigation was approved by the University of Pennsylvania Institutional Review Board (IRB), which served as the IRB of record for this trial. Prior to participation, all participants provided written consent by signing an IRB approved document indicating that they understood the trial protocol and agreed to participate. In addition to receiving cigarettes in experimental packaging at no cost, participants were paid up to $235 for completing the full trial and adhering to all elements of the protocol. No adverse events occurred during the trial.

### Participants and procedure

Sample size was determined by power analysis using G*Power [28], see S1 Protocol. Our primary outcome of interest in this trial was quit intentions. Based on past experimental investigations exploring the impact of graphic warning labels on quit intentions [6], we predicted an effect size of .35. Sample size estimates were calculated for 80% power and a two-tailed alpha of .05. After collapsing from 5 to 3 conditions as described below, we estimated that we would need 260 participants to identify a direct effect of graphic vs text-only warnings condition on quit intentions using unadjusted analysis of variance (ANOVA).

Study participants were 293 adult smokers (*M*_Age_ = 33.68, *SD* = 11.56, 45% female) recruited through fliers and ads placed on social media websites in Columbus, OH (N = 145) and Philadelphia, PA (N = 148). Recruitment was conducted from January 2013 and February 2014. All follow-up was completed by April, 2014. An initial phone screening was used to exclude individuals who smoked <5 cigarettes or >two packs per day, reported being *likely* or *very likely* to quit smoking in the next 30 days, had a contra-indicated medical condition (e.g., pregnancy), were currently being treated for a psychiatric condition or who reported a history of substance abuse. Complete inclusion and exclusion criteria can be found in S1 Protocol. Most participants identified themselves as being White (62%), 31% as Black, and 5% as more than one race. Remaining participants identified as American Indian (*n* = 3), Asian (*n* = 2) or “other” (*n* = 1). Participants reported smoking an average of 16.99 (*SD* = 7.87) cigarettes per day, and they reported having been a smoker for 17.14 (*SD* = 12.04) years. Nicotine dependence was assessed by the Fagerström Test of Nicotine Dependence [29]. The mean score was 4.37 (*SD* = 1.78) indicating moderate dependence, which was biochemically supported by breath carbon monoxide (CO) sample at intake (*M* = 17.93 ppm, *SD* = 10.63)[29].

Participants were stratified on the basis of gender, amount smoked, and quit intentions, then randomly assigned to conditions by research assistants. The randomization scheme was generated by an individual who did not interact with participants. The protocol was open label; thus no attempts at blinding were made for experimenters or participants. No differences existed between conditions on demographic characteristics or baseline smoking behaviors at randomization (see S1 Table). Depending on their assigned experimental condition, participants received their own brand of cigarettes with packages modified by text-only warning labels, graphic warning labels, or graphic warning labels that included elaborated text. Early participants (n = 117) were randomized to one of 5 conditions at a 1:1:1:1:1 ratio. The presence vs. absence of a quitline number was manipulated in conditions with images. No participant ever called the quitline. Thus, the quitline number was dropped and participants who had been exposed the quitline number were analyzed as members of the parent condition (graphic or elaborated). This change in the protocol, which was approved by the funding agencies, enabled us to recalculate the power analysis and recruit fewer participants (see S1 Protocol). For the remainder of the study, participants were randomly assigned to one of the three experimental conditions. The randomization scheme was adjusted to increase the likelihood that later participants would be included in the text-only condition, resulting in a 1:1:1 ratio across conditions for the study.

**Fig 1:**
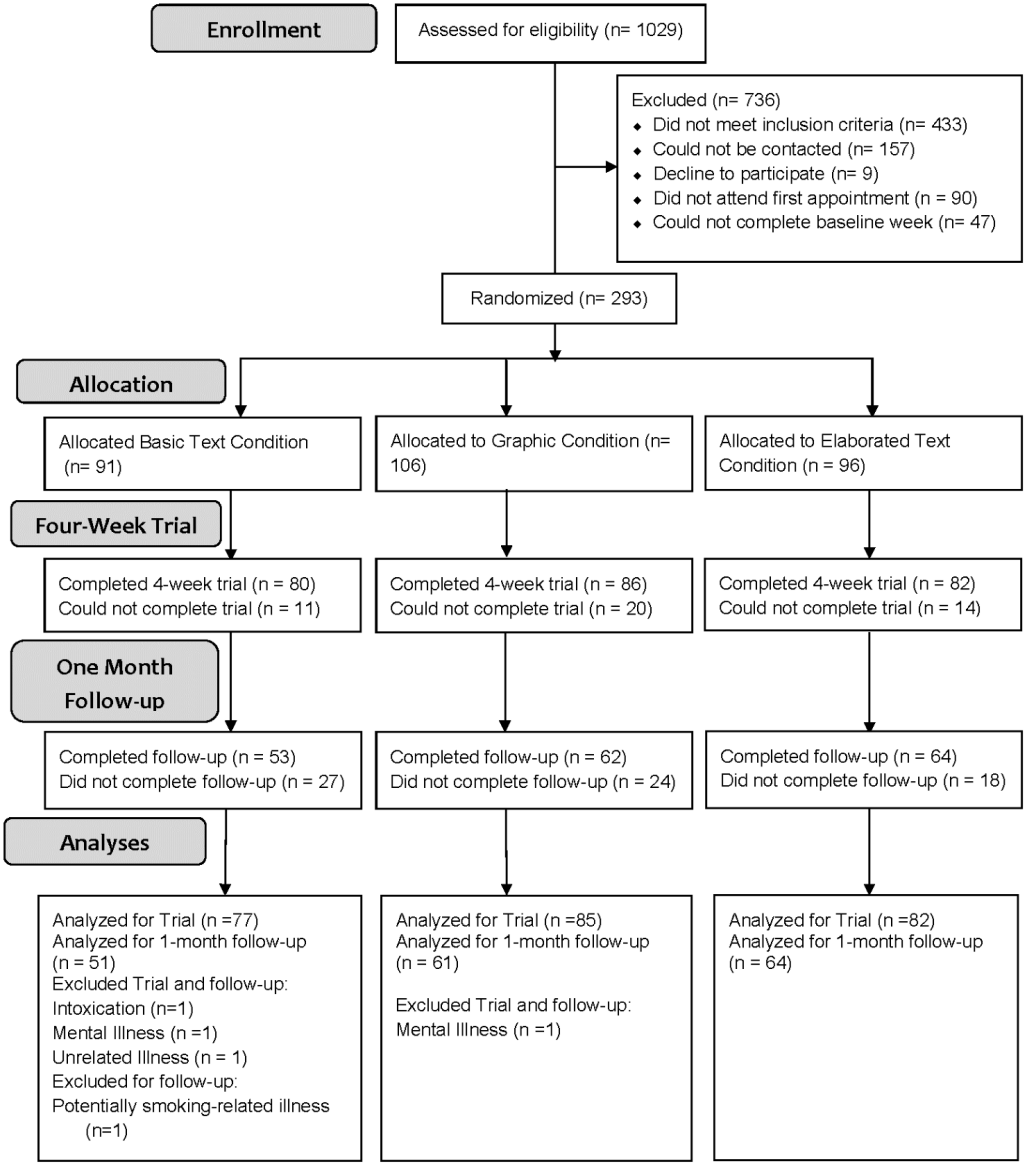
Participant Retention Diagram. Participant recruitment and retention over the course of the trial.

Participants returned to the lab each week to receive additional cigarettes and respond to surveys about their experiences with the new packaging. Participants returned any unused cigarettes from the prior week at each appointment. To reduce possible demand characteristics from exposure to cigarette packages, they completed all dependent measures at a private computer work station with no cigarette packages within sight. At the same time, a research assistant prepared cigarettes for the next week in an adjoining room. Retention was high at the end of the trial (84.6%). At that time, participants completed key dependent measures on a computer. About one month later, experimenters were able to contact most participants (60.8%) by phone for a voluntary follow-up survey, Fig 1. Further details of the experimental protocol can be found in S1 Protocol.

### Independent variable

#### Graphic Warning Manipulation

**Fig 2:**
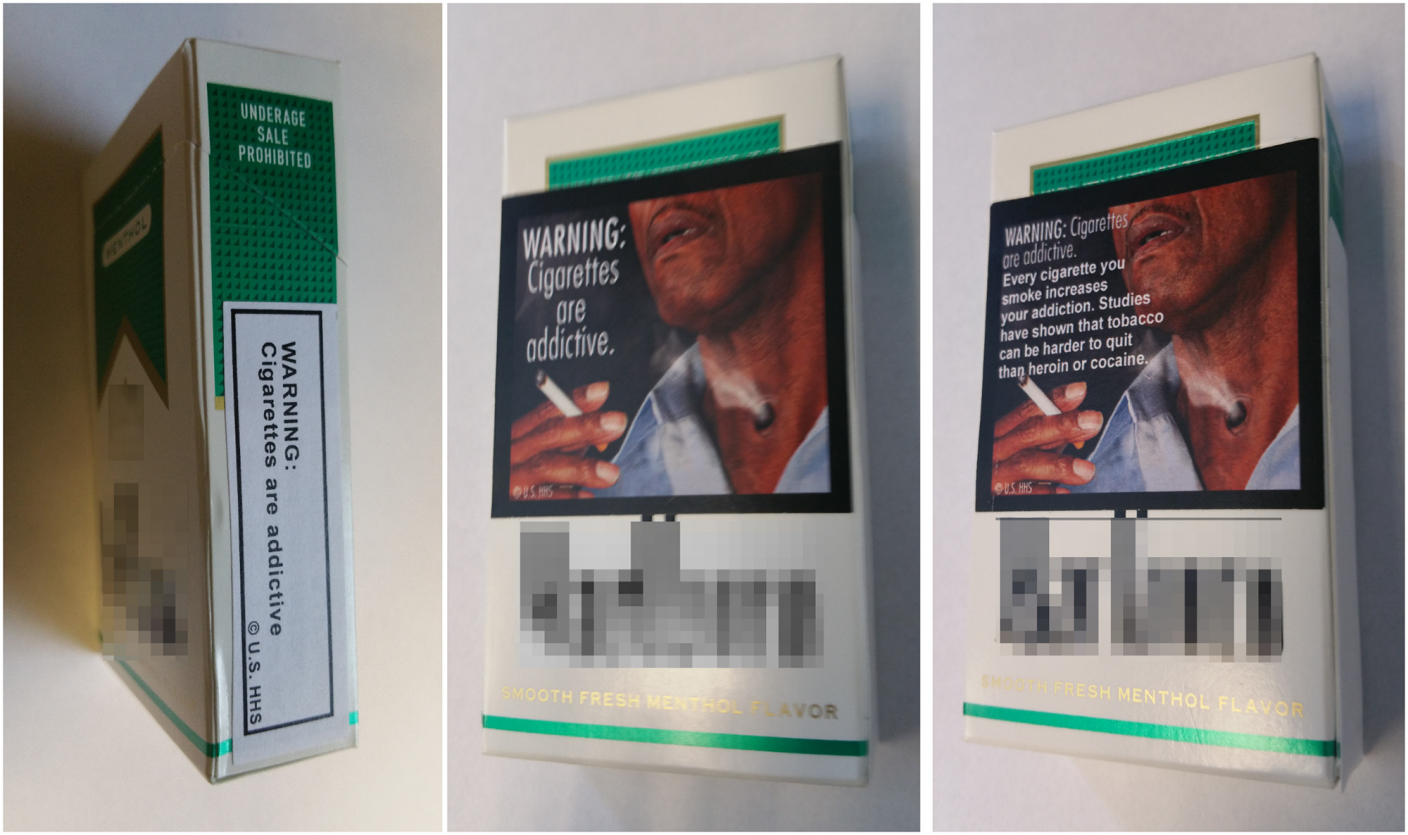
Placement of Experimental Warning Labels. Basic text warnings (left) were placed on the side of cigarettes packages. Graphic warning labels (center) covered approximately 50% of the front of cigarette packages and paired images with basic text statements. Elaborated text warning labels (right) also featured graphic images, but included descriptive text which explained the warning in more detail.

Each week, participants were given a one-week supply of their preferred brand of cigarettes. In the first week, participants were given cigarettes in non-modified packages based on self-reported amount smoked. In subsequent weeks, participants were given cigarettes in modified packages based on the amount they smoked the previous week as measured by cigarette filters returned at each lab visit [30] (see S1 Protocol for additional details). For four weeks, warning labels appropriate to their randomized experimental condition were affixed to each pack. All participants received the same nine basic text messages (e.g., “Cigarettes cause fatal lung disease.”) mandated by the Family Smoking Prevention and Control Act. Participants in the text-only condition received packages of cigarettes modified with only this basic text information, which was placed on the side of each package (see Fig 2 for examples in all three conditions; for full materials see S1–S3 Figs). Participants in the graphic image condition received the same basic text plus related images; these warnings covered approximately 50% of the front and back of each pack (placed so as not to cover brand information). These graphic labels featured the nine images proposed by FDA for inclusion on US cigarette packs [26]. Participants in the elaborated text condition received packs with warnings covering the same package space; these labels included the same text and images plus additional text describing how every cigarette entails risk (e.g., “Every cigarette you smoke increases your risk of crippling, often fatal, lung diseases such as emphysema.”). Participants were exposed to each label in their condition in approximately equal proportions over the experimental period.

### Dependent measures

#### Smoking behavior

To monitor smoking behavior over the course of the trial, participants were required to return their used cigarette filters to the lab each week [30]. Participants were provided with a resealable plastic pouch for each day between laboratory sessions. Returned filters were counted to measure smoking behavior. To estimate smoking behavior before packaging was manipulated, the number of filters returned each day during the baseline period was averaged. To estimate smoking behavior during the trial, the number of filters returned each day during the modified warning labels period was averaged.

#### Negative affect

During their third lab visit, after using cigarettes in modified packaging for one week, participants were asked to indicate the extent to which the cigarette packaging affected their feelings about smoking (“Did the packaging change how you feel about smoking?” 1 = the packaging made me feel much worse about smoking; 4 = the packaging had no effect; 7 = the packaging made me feel much better about smoking). This item was adapted from past research [7] and reverse coded, so that higher scores indicated more negative affect.

#### Risk scrutiny

Scrutiny of risk information was also assessed during the third lab visit using a measure adapted from past research on warning label scrutiny [31]. Participants were asked “In the past week, have you read or looked closely at the information on your cigarette packs?” and answered on a 5-point scale (1 = *not at all* to 5 = *extremely often*).

#### Warning credibility

Perceptions of warning credibility were assessed after four weeks of exposure to the experimental warning labels, during the sixth and final lab visit. After completing all other dependent measures, participants viewed each warning label that had been on their cigarette packages during the study and responded to “Do you believe that what this information says is true?” (1 = *not at all* to 7 = *completely*). This measure was adapted from past research on perceptions of cigarette warning label credibility [13] and used to construct an index of perceived warning credibility by averaging ratings across labels (Cronbach’s α = .92).

#### Risk perceptions

Subjective perceptions of smoking illness risk were assessed on three 7-point scales adapted from past research on smoking risk perceptions [32, 33] during the final lab visit. Comparative risk perceptions were assessed with “Compared to the average person your age, gender, and race, how would you rate your chances of getting a life-threatening illness because of smoking?” (1 = *much lower* to 7 = *much higher*). Absolute risk perceptions were assessed with two items: “If I continue to smoke, I think my chances of getting a life-threatening illness are” (1 = *almost zero to 7 = almost certain*) and “If I continue to smoke, I would feel very vulnerable to dying at a younger age than I would otherwise” (1 = *strongly disagree to* 7 = *strongly agree*).

#### Quit intentions

During their final lab visit, participants responded to three items relevant to quit intentions. These items included a contemplation ladder [34], which asked them to choose the number that indicates their current thinking about smoking on a 9-point scale (1 = taking action to quit [e.g., cutting down, enrolling in a program]; 3 = starting to think about how to change my smoking patterns; 5 = think I should quit but not quite ready; 7 = think I need to consider quitting someday; and 9 = no thought of quitting). This item was reverse-scored. Participants also responded to two 4-point scale items adapted from past research [35], which asked “How likely do you think it is that you will try to quit smoking within the next 30 days?” (1 = *very unlikely* to 4 = *very likely*) and “How much do you want to quit smoking?” (1 = *not at all* to 4 = *a lot*).

#### Label memory

Consistent with procedures used in past research to investigate memory for emotional events, participants were asked to recall the warning labels during their final laboratory session in the trial [6, 36]. An open-ended item asked “Please think about the packaging of the cigarettes we have given you over the past few weeks. Please try to recall what the information on the packages stated and write it in below.” Participants were given nine text boxes and no time limits to list as much information as they could recall. Four independent coders evaluated responses for evidence of the presence of memory for the components of each label (basic text/images/ elaborated text). Although lung cancer and throat cancer were not mentioned explicitly on the warning labels, participants often inferred these illnesses from information on the labels. Thus, any mention of lung or throat cancer was included as memory for label content, but was not classified as memory for any specific label element. This resulted in a total possible score of 29. Inter-rater reliability was established for memory of each label component (mean Krippendorff’s α = .95) and the number of components recalled (Krippendorff’s α = .96).

#### Smoking risk knowledge

Participants’ knowledge of smoking-related illnesses also was measured through a free response task at the end of the trial. Some researchers believe that free response measures are better indicators of smoking risk knowledge than responses to closed ended questions [37]. Participants responded to the item “What are some diseases you’ve heard about that can be related to tobacco use?” Participants were given a large text box in which to enter as many diseases as they could think of. Two independent coders examined these responses and coded for any mention of 29 different smoking-related health risks. Responses were coded for widely known smoking-related illnesses (e.g., lung cancer; heart disease) and less widely known risks (e.g., breast cancer; joint problems). Inter-rater reliability was high (Krippendorff’s α = 1.00). Risk knowledge was calculated by summing the number of smoking-related illnesses mentioned (possible range = 0–29).

During a telephone follow-up one month later, participants were asked again to verbally list as many smoking-related diseases as they could. These responses were recorded by research assistants and again coded by two independent coders for the presence of 29 different smoking-related illnesses. Inter-rater reliability was high (Krippendorff’s α = 1.00).

#### Analysis Strategy

Prior to our primary analyses, one-way analysis of variance (ANOVA) and chi-square (χ^2^) tests were used to investigate the possibility that participant demographic characteristics or baseline smoking behaviors might differ as a function of experimental condition at randomization and in the data we analyzed. We also used unadjusted one-way ANOVAs to investigate the possibility that participant exposure to the warning information might have differed as a function of experimental condition and that warning label exposure may have had direct effects on key outcome variables.

Consistent with an intention to treat analysis strategy, available data for participants who completed the trial were analyzed as if they had complied with the intervention condition to which they were randomly assigned, regardless of compliance with the experimental protocol. All analyses were conducted after the end of the trial and follow-up data collection. To test hypotheses about the indirect (mediated) effects of exposure to graphic warning labels, we used structural equation modeling (SEM). Analyses were conducted using MPlus Version 7 [38]. We created two orthogonal contrast-coded predictors to represent the effects of the two warning conditions. One predictor contrasted the two graphic conditions (coded as 1) versus text-only warnings (coded as -2). The other contrasted the elaborated text condition (coded as 1) versus the graphic-only condition (coded as -1), with the text-only condition coded as 0. We used these contrast codes to fit models based on our theoretically derived predictions about the indirect effects of graphic warning labels on risk perceptions, quit intentions, and smoking risk knowledge. In the structural equation models, a pairwise present approach was taken to handling missing data for participants who completed the trial. This approach uses all available data about variable relationships in estimating model parameters.

To assess the possibility that non-normal distribution could influence results, all analyses were first conducted using maximum likelihood estimation with robust standard errors, an approach to structural equation modeling that is robust to non-normally distributed data [39]. Modification indices were used to identify plausible paths not predicted by our theoretical framework. Satorra-Bentler scaled χ^2^ comparison tests were computed to compare nested models [40]. Models which evidenced acceptable fit were recalculated using bootstrap resampling. We used several different indices to determine the fit of each structural equation model including the normed χ^2^ and the relative χ^2^ [41]. A non-significant χ^2^ and a relative χ^2^ of less than 2 indicate good model fit [41]. We also report the root mean squared error of approximation (RMSEA). RMSEA values below .05 are considered to indicate that the model fits the data well [42]. Finally, we report the Comparative Fit Index (CFI), and the Tucker-Lewis Index (TLI). CFI and TLI values above .95 are considered indicators of good model fit [42]. For the purpose of comparing non-nested models, we also report the Akaike information criterion (AIC) and Bayesian information criterion (BIC). The significance of indirect (mediated) effects was assessed by using bootstrap re-sampling procedures, which used 5,000 random samples drawn from the existing data with replacement to generate bias-corrected confidence intervals for each indirect effect [43]. Confidence intervals which do not contain zero indicate a significant indirect effect. Bootstrap resampling is well suited to estimating indirect effects in multiple mediator models because confidence intervals are based on the sampling distribution of the indirect effect rather than an assumed normal sampling distribution [44, 45].

To address the possibility that differential attrition based on demographic characteristics or smoking behavior might bias our results, we used logistic regressions and χ^2^ tests to compare the characteristics of participants randomized to experimental conditions who did vs. did not complete the trial. Individual differences that were related to attrition were used to create imputation models from which we imputed missing data and retested each of our hypotheses. To do so, we created ten data sets using multiple imputation with chained equations (MICE) in Stata version 12 [46]. Structural equation models were then calculated from the imputed data sets using the MPlus multiple imputation analysis procedures.

Finally, to further assess the validity of our theoretically derived model described in hypotheses 1 and 2, we also tested the fit of the data to two plausible alternative models and report their fit statistics. We compared the fit of the data to these models to the fit of the data in our hypothesized model using the AIC and BIC.

## Results

In our primary analyses, participants who did not complete the week six appointment were excluded from analyses (n = 45). Retention did not differ as a function of experimental condition. Four additional participants were excluded from analyses (two were hospitalized for mental illness during the trial, one was diagnosed with a possible smoking-related illness during the trial, and one reported intoxication during lab sessions), leaving 244 participants at week 6. Follow-up data were excluded for one additional participant who was diagnosed with a possible smoking related illness after completing the trial. Demographics did not differ by experimental condition at random assignment (see S1 Table) or at the end of the trial, Table 1. Additionally, previous research does not identify demographic differences as moderators of the impact of graphic warnings labels [47]. Therefore, reported one-way ANOVAs do not control for demographic variables. All reported structural equation models controlled for the demographic variables used in stratified randomization. Specifically, all reported structural equation models control for gender (female vs. male), baseline smoking heaviness (less vs. more than 20 cigarettes per day), and baseline quit intentions (*1* “very unlikely” vs. *2–4* “very likely”).

**Table 1:** Demographics of included participants by experimental condition at week 6.

	Text-only (N = 77)	Graphic Images(N = 85)	Graphic Images and Elaborated Text (N = 82)	Test Statistic, p-value
**Age**				
	32.78 (11.57)	34.98 (11.59)	35.12 (11.96)	F(2, 241) = .99, p = .37
**Gender**				
Male	40	47	46	χ² (2) = .35, p = .84
Female	37	37	36	
Other	0	1	0	
**Race**				
White	45	53	48	χ² (4) = 3.39, p = .49
Black	26	24	31	
Asian	1	1	0	
American Indian	2	1	0	
More than one	2	5	3	
Other	1	1	0	
**Ethnicity**				
Hispanic	2	3	3	χ² (2) = .17, p = .92
Non-Hispanic	75	82	79	
**Education**				
Some high school, no degree	6	6	6	χ² (8) = 6.31, p = .61
High school degree or GED	21	16	24	
Some college, no degree	29	43	31	
Associate’s Degree	9	7	5	
Bachelor’s Degree	12	11	12	
Master’s Degree or higher	0	1	2	
**Self-report Cigarettes Smoked Daily**				
	15.79 (6.94)	16.82 (7.82)	16.40 (7.81)	F(2, 241) = .38, p = .69
**Years of Smoking**				
	16.52 (11.81)	18.15(12.60)	17.88(12.15)	F(2, 241) = .41, p = .67
**Fagerström Test of Nicotine Dependence**				
	4.51 (1.77)	4.27 (1.94)	4.32 (1.85)	F(2, 241) = .36, p = .70
**Breath Carbon Monoxide (CO)**				
	17.01 (10.13)	19.45 (13.02)	18.99 (9.68)	F(2, 241) = 1.09, p = .34

Due to a low number of participants in certain groups, some categories were combined in χ^2^ tests for demographic differences after random assignment. The participant who indicated their gender as “other” was excluded from the χ^2^ test for gender. Participants who indicated their race as Asian, American Indian, More than one race, or Other, were combined to form a single “other” category. Finally, participants with a Master’s degree or higher were combined with participants who held a Bachelor’s degree to form a single high-education category.

Participant smoking behavior did not differ significantly by experimental condition during the baseline period, *F*(2, 241) = .62, *p* = .538, or during the trial period, *F*(2, 241) = .79, *p* = .454, Table 2. On average, participants were exposed to the warning labels over 15.7 times per day across conditions during the trial. A paired samples t-test comparing the rates of smoking at baseline and during the trial revealed a trend indicative of a slight increase in smoking behavior after the baseline period *t*(243) = 1.86, *p* = .064, which may be the result of participants acclimating to receiving experimental cigarettes at no cost.

**Table 2:** Average smoking behavior by experimental condition at baseline and during the trial.

	Text-only	Graphic Images	Graphic Images and Elaborated Text	Omnibus Effect Size
	*Mean* (*SD*)	*Mean* (*SD*)	*Mean* (*SD*)	*η*_*p*_^*2*^
**Filters returned during baseline**	14.76 (6.41)	16.01 (7.22)	15.42 (7.63)	.01
[CI 95]	[13.95, 15.56]	[15.1, 16.91]	[14.46, 16.38]	
**Filters returned during the trial**	14.85 (6.93)	16.29 (7.55)	15.95 (8.11)	.01
[CI 95]	[13.97, 15.72]	[15.34, 17.24]	[14.93, 16.97]	

Due to a computer error, 30-day quit intentions were not recorded for 22 participants who completed the trial. Because 30-day quit intentions were also measured at week 5 and the week 5 and week 6 responses were highly correlated (*r* = .76, *p* < .001), we carried forward week 5 quit intention responses to week 6 for participants missing this item only. Model fit, path coefficients, and indirect effects were comparable when these participants were excluded list-wise.

One-way ANOVAs revealed that the graphic warning conditions (compared to text-only) increased negative affect toward smoking, scrutiny of the risks, and label memory. The graphic-only condition increased warning credibility relative to the other two conditions. There were no significant differences between conditions in risk perceptions, quit intentions, or risk knowledge, Table 3.

**Table 3:** Unadjusted mean outcome responses by experimental condition among participants who completed the trial.

	Text-only	Graphic Images	Graphic Images and Elaborated Text	Omnibus Effect Size
	*Mean* (*SD*)	*Mean* (*SD*)	*Mean* (*SD*)	*η*_*p*_^*2*^
**Negative Affect**	4.52^A,B^ (.87)	5.30^A^ (1.14)	5.09^B^ (1.27)	.08
[CI 95]	[4.27, 4.72]	[5.05, 5.54]	[4.84, 5.32]	
**Risk Scrutiny**	3.32^A,B^ (1.30)	3.70^A^ (1.19)	3.84^B^ (1.07)	.03
[CI 95]	[3.03, 3.62]	[3.44, 3.96]	[3.60, 4.08]	
**Perceived Credibility**	6.16^A^ (1.14)	6.51^A,B^ (.70)	5.99^B^ (1.17)	.04
[CI 95]	[5.90, 6.42]	[6.36, 6.66]	[5.73, 6.25]	
**Quit Intentions (Standardized)**	0.11 (.92)	-0.01 (.88)	-0.09 (.89)	.01
[CI 95]	[-.10, .32]	[-.20, .18]	[-.28, .11]	
**Risk Perceptions (Average)**	5.20 (1.29)	5.41 (1.27)	5.19 (1.38)	.01
[CI 95]	[4.90, 5.50]	[5.13, 5.69]	[4.89, 5.50]	
**Label Memory at Week 6**	3.92^A,B^ (1.36)	4.98^A^ (1.79)	5.13^B^ (2.31)	.08
[CI 95]	[3.61, 4.23]	[4.59, 5.36]	[4.61, 5.64]	
**Risk Knowledge at Week 6**	3.61 (1.53)	4.09 (1.96)	3.88 (1.72)	.01
[CI 95]	[3.26, 3.96]	[3.66, 4.52]	[3.50, 4.27]	
**Risk Knowledge at Follow-up**	3.55(1.32)	3.50 (1.54)	3.67(1.64)	.00
[CI 95]	[3.18, 3.92]	[3.10, 3.90]	[3.26, 4.08]	

Shared superscripts indicate that values are significantly different, *p* < .05. Direct effects of condition on Risk Perceptions and Quit Intentions are based on average responses to relevant measures. An index of Quit Intentions reported in this table was created by averaging standardized responses to each of the three quit intention measures (α = .88). An index of Risk Perceptions were created by averaging across the three risk perception measures (α = .70).

### Hypotheses 1 and 2

We proposed that graphic warning labels would influence risk perceptions, and quit intentions in turn, through multiple affective paths. To ensure that risk perceptions and quit intentions were sufficiently distinct constructs, we compared measurement models with one vs. two factors. The single factor model did not fit the data well, as evidenced by a highly significant χ^2^ (χ^2^ [9] = 90.50, *p* < .001), high RMSEA (.193), and low CFI (.861) and TLI (.769) values. A two-factor model offered a better fit, (χ^2^[8] = 11.93, *p* = .155; RMSEA = .045, CFI = .99; TLI = .99). A χ2 difference test for nested models revealed that the two-factor solution fit the data better than the one-factor model (χ^2^_Diff_ = 78.57, *p* < .001). Thus, a two factor measurement model was appropriate for tests of Hypotheses 1 and 2. We predicted that the presence of graphic images would increase negative affect toward smoking, which would increase risk perceptions and quit intentions directly and indirectly by encouraging smokers to think about and form favorable evaluations of the risk information warnings provided. A model testing these predictions using maximum likelihood estimation with robust standard errors fit the data well (χ^2^[59] = 65.59, *p* = .259, relative χ^2^ = 1.11, RMSEA = .02 [CI 90: .00 to .05], CFI = .99, TLI = .99). However, modification indices suggested that including a path from negative affect to perceived credibility would improve model fit. A model with this path was estimated using maximum likelihood estimation with robust standard errors and also fit the data well (χ^2^[58] = 61.52, *p* = .351, relative χ^2^ = 1.06, RMSEA = .02 [CI 90: .00 to .04], CFI = 1.00, TLI = .99). A Satorra-Bentler scaled χ^2^ difference test revealed that the alternative model provided a marginally better fit to the data than the hypothesized model, χ^2^[1] = 3.70, *p* = .056). Therefore, this path was retained in our final model, which was retested using bootstrap resampling to calculate confidence intervals for indirect effects. Modification indices did not suggest any additional paths to be added or removed from the model.

**Fig 3:**
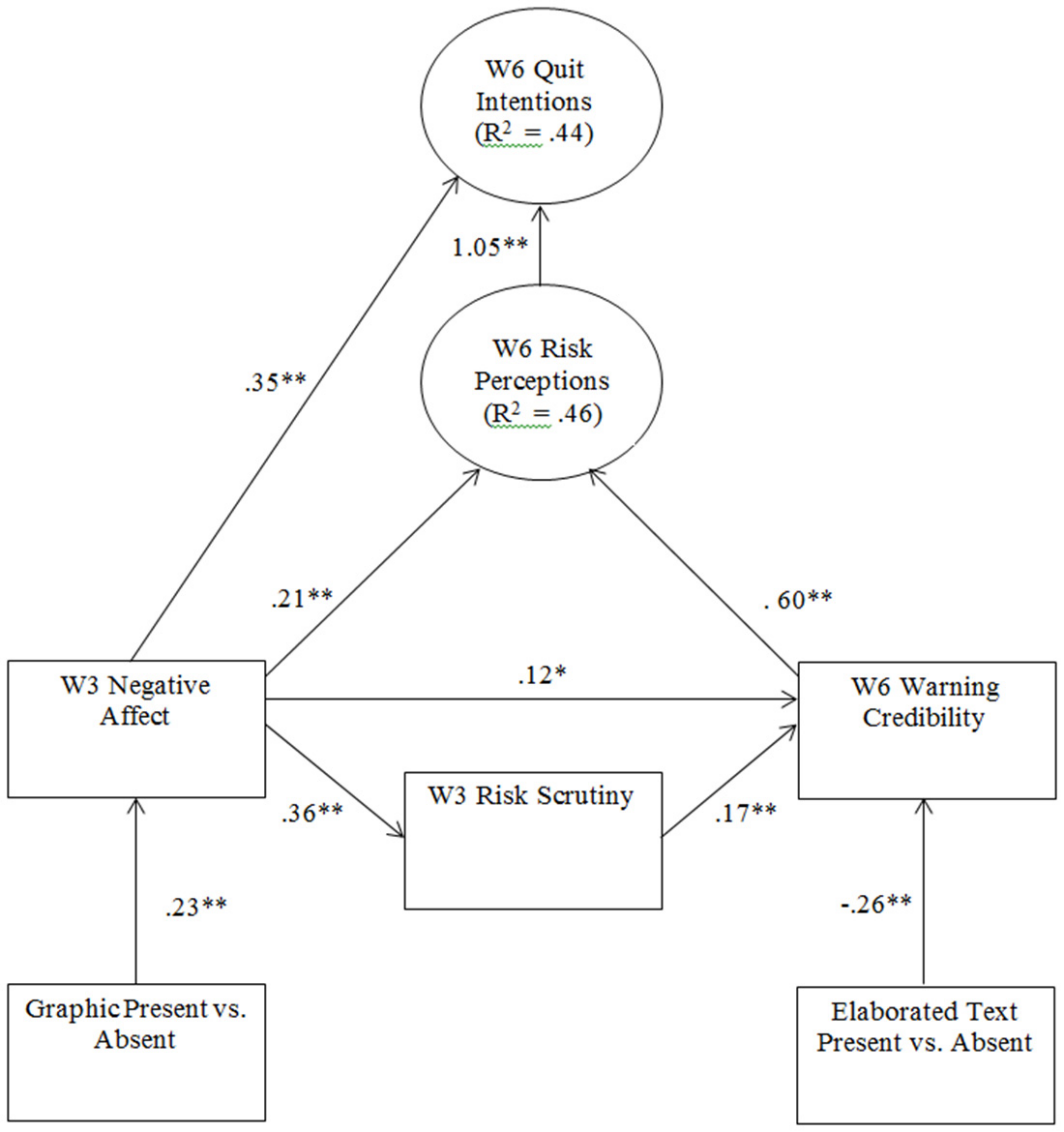
Model Testing the Predictions of Hypotheses 1 and 2. W3 = Week 3; W6 = Week 6. Path coefficients for the measurement models for Risk Perceptions (Risk 1 = 1.00, Risk 2 = .88**, Risk 3 = 1.19**) and Quit Intentions (Contemplation Ladder = 1.00, 30-Day Quit Intentions = .45**, Quit Desire = .42**)

The final model testing Hypotheses 1 and 2 is depicted in Fig 3. The recalculated model with bootstrap resampling also fit the data well, as evidenced by a non-significant χ^2^ (χ^2^[58] = 62.94, *p* = .306), a relative χ^2^ of less than 2 (1.09), a low RMSEA (.02, CI 90: .00, .05), and high CFI and TLI values (.99 and .99). The similarity of these results to the results of the same model estimated with MLR suggests that non-normal variable distributions did not significantly bias the trial’s results. The AIC for this model was 8215.82 and the BIC was 8397.46. As predicted by H1a, warning labels with graphic images elicited more negative affect toward smoking than text-only warning labels at week 3 (*b* = .23, *p* < .001). Consistent with the affect heuristic, this led to heightened risk perceptions (*b* = .21, *p <* .001); negative affect also led to increased quit intentions independent of risk perceptions at week 6 (*b* = .35, *p* = .004). As predicted by H1b, negative affect also led to increased risk scrutiny at week 3 (*b* = .36, *p* < .001), which then led to heightened perceptions of warning credibility at week 6 (*b* = .17, *p* = .009). There was also a direct link between negative affect and heightened perceptions of warning credibility at week 6 (*b* = .12, *p* = .033). As predicted by H1b, warning credibility was a significant predictor of risk perceptions at week 6 (*b* = .60, *p* < .001). Finally, heightened risk perceptions predicted increased week 6 quit intentions (*b* = 1.05, *p* < .001). Contrary to H2, graphic warning labels with elaborated text were perceived as less credible at week 6 than graphic warnings with basic text only (*b* = -.26, *p* = .001).

All indirect paths in the final model were statistically significant, Table 4. The positive indirect effect of graphic presence -> negative affect ->risk perceptions ->quit intentions is consistent with the negative affect aroused by the presence of graphic images acting as a cue from which people infer risk (i.e., the Affect Heuristic [8]), which in turn leads to quit intentions. The positive indirect effect of graphic presence -> negative affect -> quit intentions indicates that affect influenced quit intentions apart from risk perception. The positive indirect effects of graphic presence -> negative affect -> risk scrutiny -> warning credibility -> risk perceptions -> quit intentions and graphic presence ->negative affect -> warning credibility -> risk perceptions -> quit intentions are consistent with images which arouse negative affect motivating more careful consideration of warning information (i.e., affect as a spotlight [11,12]). Finally, the negative indirect effect of elaborated text presence -> perceived credibility -> risk perceptions -> quit intentions suggests that the impact of affect on risk perceptions and quit intentions only occurs when people find the warnings credible, which was not the case with the warnings that included elaborated text.

**Table 4:** Indirect effects of condition on risk perceptions and quit intentions.

Mediation Path	Indirect Effect (SE)
Affect Heuristic path (H1a): Graphic Present vs. Absent -> Negative Affect -> Risk Perceptions -> Quit Intentions	**.050 (.019)**
[CI 95]	**[.019, .094]**
Affect Motivates Quit Intentions: Graphic Present vs. Absent -> Negative Affect -> Quit Intentions	**.079 (.030)**
[CI 95]	**[.021, .140]**
Affect as Spotlight Motivates Greater Scrutiny of Message (H1b): Graphic Present vs. Absent -> Negative Affect -> Risk Scrutiny -> Warning Credibility -> Risk Perceptions -> Quit Intentions	**.009 (.005)**
[CI 95]	**[.002, .020]**
Affect Enhances Warning Credibility: Graphic Present vs. Absent -> Negative Affect -> Warning Credibility -> Risk Perceptions -> Quit Intentions	.018 (.010)
[CI 95]	**[.021, .140]**
Elaborated text and Warning Credibility (H2): Elaborated Text Present vs. Absent -> Warning Credibility -> Risk Perceptions -> Quit Intentions	**-.165 (.056)**
[CI 95]	**[-.289, -.082]**
Total indirect effect of Graphic Present vs. Absent on Quit Intentions	**.155 (.038)**
[CI 95]	**[.085, .234]**
Total indirect effect of Elaborated Text Present vs. Absent on Quit Intentions	**-.165 (.056)**
[CI 95]	**[-.289, -.082]**

Bold indicates a reliable indirect effect, where *p* < .05.

### Investigating the influence of attrition

Participants who completed the trial were older (*b* = 2.21, *p* = .028) and lighter smokers (*b* = 3.27, *p* = .001) than participants who did not complete the trial. Race, ethnicity, education, and other smoking characteristics were not significant predictors of attrition (for details of these analyses, see S2 Table). Participant age, self-reported number of cigarettes smoked daily, and all variables shown in Fig 3 were used to impute data for participants who were randomized to experimental conditions but did not complete the study and for items participants who completed the study declined to answer. Covariates were not included in the imputation model because the “smoking heaviness” covariate was derived from self-reported number of cigarettes smoked daily at baseline. These full data sets (n = 289) were then used to test the model presented in Fig 3. Control variables were included when imputed data were fit to the hypothesized model. The model fit the imputed data well, as evidenced by a non-significant χ^2^ (χ^2^[58] = 61.93, *p* = .338), a relative χ^2^ of less than 2 (1.07), a low RMSEA (.02, CI 90: .00, .04), and high CFI and TLI values (1.00 and .99). All model paths remained significant, S4 Fig.

### Testing alternative models

Although our hypothesized model fit the data well, we also tested two additional models derived from the predictions of alternative theoretical frameworks. Some researchers have suggested that graphic warning labels are effective primarily because they are more salient than text-only warnings [43]. In this account, image salience causes smokers to scrutinize risk information, and risk scrutiny increases negative affect, risk perceptions, and quit intentions [48]. We tested an alternative model consistent with this account, which reversed the order of risk scrutiny and negative affect. The data did not fit this model as well as our final model, χ^2^(58) = 98.84, *p* < .001, relative χ^2^ = 1.70, RMSEA = .05 (CI 90: .04 to .07), CFI = .95, TLI = .93. The AIC (8251.73) and BIC (8433.37) were larger for this model than our hypothesized model (AIC_Difference_ = 35.91; BIC_Difference_ = 35.91), which further suggests that the data did not fit this model as well as it fit our hypothesized model. Path coefficients for this model are available to interested readers in S5 Fig.

Self-perception theory, derived from the psychological literature on attitude change, suggests that people often infer their beliefs from their decisions [49]. Within the context of graphic warnings labels, this perspective would suggest that smokers may infer their risk perceptions from observing their stated quit intentions. We tested an alternative model in which risk perceptions were predicted by quit intentions rather than the reverse. All paths in this alternative model were identical, except the location of the two latent constructs was reversed, such that quit intentions were predicted by negative affect and warning credibility and risk perceptions were predicted by negative affect and quit intentions. The data did not fit this model as well as our final model, χ^2^(58) = 104.49, *p* < .001, relative χ^2^ = 1.80, RMSEA = .06 (CI 90: .04 to .08), CFI = .94, TLI = .92. The AIC (8257.38) and BIC (8439.02) were larger for this model than our hypothesized model (AIC_Difference_ = 41.56; BIC_Difference_ = 41.56), which further suggests that the data did not fit this model as well as it fit our hypothesized model. Path coefficients for this model are available to interested readers in S6 Fig.

After examining two plausible alternative models derived from past research, we concluded that the model which tested Hypotheses 1 and 2 fit the data comparatively better. This suggest that the present paper’s theoretical conception of how graphic warning labels influence smokers’ risk perceptions and quit intentions provides a better account of the data in this randomized clinical trial than the alternative theories. Further, these analyses suggest that the temporal relationships we hypothesized provide a better explanation for the data than alternative relationships.

### Hypothesis 3

We proposed that graphic warning labels would be more memorable than text-only warnings (due to the increased negative affect towards smoking or to the presence of more detailed risk information), and that this increased memory for warning information would lead to greater smoking risk knowledge. Existing literature suggests that increased memory for warning information could be driven by the negative affect aroused by images. However, in the current research, negative affect was not correlated with label memory or risk knowledge, Table 5, precluding the possibility that negative affect mediates the impact of graphic images on memory. The second contrast comparing the presence of elaborated text (1) to the other two warning conditions (-1) was also uncorrelated with memory for warning information (see Table 5). Thus, we used the contrast of the two graphic conditions (coded as 1) with the text-only warnings (coded as -2) to investigate the impact of images on label memory on risk knowledge immediately after completing the trial and at follow-up.

**Table 5:** Correlations among key measures.

	1.	2.	3.	4.	5.	6.	7.	8.	9.	10.
1. Graphic Present vs. Absent	-									
2. Elaborated Text Present vs. Absent	-.01	-								
3. Negative Affect	.**27** **	-.08	-							
4. Risk Scrutiny	.**17** **	.05	.**34** **	-						
5. Perceived Credibility	.04	-.**21** **	.**22** **	.**24** **	-					
6. Mean Risk Perceptions	.04	-.07	.**32** **	.**23** **	.**55** **	-				
7. Mean Quit Intentions	-.08	-.04	.**36** **	.**16** *	.**37** **	.**48** **	-			
8. Label Memory at Week 6	.**27** **	.03	.09	.10	.08	.10	.04	-		
9. Risk Knowledge at Week 6	.10	-.05	.02	.04	.07	.04	.10	.**16** *	-	
10. Risk Knowledge at Follow-up	.01	.05	.02	.02	-.07	-.01	-.01	.**17** *	.**43** **	-

*p < .05.

**p < .01.

Mean Quit Intentions include scores with week 5 values carried forward for the 22 data points where week 6 quit intentions were not recorded due to computer error.

When estimated using maximum likelihood with robust standard errors, the model which examined graphic image presence -> label memory at week 6 -> risk knowledge at week 6 provided an adequate fit to the data as evidenced by a non-significant χ^2^ (1) = 1.525, *p* = .217, a relative χ^2^<2 (1.525), a low RMSEA (.047, CI 90: .000, .185), and high a CFI (.98). Unexpectedly, the TLI value was below the threshold for good model fit (TLI = .85). Fit statistics were similar when this model was rerun using bootstrapping (χ^2^[1] = 1.389, *p* = .239, relative χ^2^ = 1.389, RMSEA = .04 [CI 90: .00 to .18], CFI = .99, TLI = .88), suggesting that non-normal variable distributions did not significantly bias results. Image presence led to increased label memory at week 6 (*b* = .37, *p* < .001), and label memory led to increased smoking risk knowledge immediately after participants had completed the trial (*b* = .14, *p* = .010). Bootstrapping analysis revealed that this indirect effect was significant (Estimated Indirect Effect = .05 [SE = .02], CI 95: .010, .097). The model explained 7.1% of differences in risk knowledge at week 6 (R^2^ = .071)

The model which examined graphic image presence ->label memory -> smoking risk knowledge at follow up (for the 169 participants who completed the follow-up) also fit the data acceptably when estimated using maximum likelihood with robust standard errors, χ^2^(1) = .07, *p* = .787, χ^2^_relative_ = .07, RMSEA = .00 (CI 90 .00 to .11), CFI = 1.00, TLI = 1.38). Model fit was similar when this model was rerun using bootstrapping, χ^2^(1) = .06, *p* = .805, relative χ^2^ = .06, RMSEA = .00 (CI 90: .00 to .11), CFI = 1.00, TLI = 1.41, suggesting that non-normal variable distributions did not significantly bias results. For this subgroup, image presence predicted label memory at week 6 (*b* = .37, *p* < .001), and label memory predicted smoking risk knowledge at follow up (*b* = .14, *p* = .025). This indirect effect was significant (Estimated Indirect Effect = .05 [SE = .02], CI 95: .008, .103). The model explained 6.6% of differences in risk knowledge at follow-up (R^2^ = .066).

#### Investigating the influence of attrition

To investigate the possibility that differences in attrition might account for the effects of label memory on smoking risk knowledge, we imputed memory data for participants who did not complete the trial or declined to respond to these items. Participant age and self-reported number of cigarettes smoked daily (both significantly associated with attrition), experimental condition, and available label memory and risk knowledge data were used to create 10 complete data sets. These full data sets (n = 289) were then used to retest the models for hypothesis 3. The χ^2^, relative χ^2^, and RMSEA suggest that the imputed data sets also provided a good fit to our model predicting smoking risk knowledge at week 6 (χ^2^[7] = 12.04, *p* = .099, relative χ^2^ = 1.72, RMSEA = .05 [CI 90: .00 to .10]). Unexpectedly, the CFI and TLI did not, CFI = .80, TLI = .75). All fit statistics suggest that the full data sets provided a good fit to our model predicting smoking risk knowledge at follow-up (χ^2^[7] = 6.53, *p* = .479, RMSEA = .00 [CI 90: .00 to .07], CFI = 1.00, TLI = 1.04). Path coefficients for these imputed models were similar in magnitude to the values in models that only included participants who completed the assessments, S7 Fig. Thus, these tests suggest that attrition does not account for the impact of graphic warnings on smoking risk knowledge.

## Discussion

The current research represents the first experimental investigation in a naturalistic setting of the psychological processes by which graphic warning labels influence smokers’ affect and cognitions regarding their habit. As such, it represents the best to-date approximation of real world impact of graphic warning labels. In the study, graphic warning labels (compared to text-only) increased negative affect toward smoking, elicited greater scrutiny of the warning message, and enhanced label memory. The graphic-only condition also increased the credibility of the warning message. In analyses of mediated effects on perceived risks of smoking and intentions to quit, we found support for H1a and H1b that multiple functions of affect influenced these outcomes [11, 12]. Warnings with graphics did, in fact, elicit more negative affect than text-only warnings, and, consistent with past research on the affect heuristic [8], more negative affect increased risk perceptions (and quit intentions in turn). Increased negative affect also motivated greater quit intentions directly. Third, it also elicited greater scrutiny of the messages and increased perceptions of warning credibility. This result is consistent with affect acting as a spotlight and motivating greater processing of the risk information consistent with that negative affect. In addition, increased credibility perceptions led to heightened risk perceptions and quit intentions. The elaborated text condition, however, did not support H2 and, instead, reduced credibility perceptions and counteracted the images’ effect on heightened risk perceptions.

Consistent with H3, the presence of graphic images also increased memory for label content, which led to greater smoking risk knowledge immediately after participants completed the trial and approximately one month later. Thus, warnings containing images also enhanced memory for label content, which helped to educate smokers about the harms of smoking. Although we predicted that negative affect from week 3 would drive increased memory for warning information at week 6 [18, 19], the data were inconsistent with this prediction. Several possible reasons exist for this lack of effect: (1) negative affect may have already started to fade after a week of exposure [24, 25], (2) the negative affect measure focused on feelings about smoking rather than feelings about the warnings themselves, and (3) the measure focused on valence rather than on the arousal linked with memory in past studies [18, 19]. The first possibility seems unlikely given that negative affect remained strong enough to have significant associations with several other key variables. Alternatively, it may be the case that negative affect is unrelated to memory for warning information. Instead, it may be that simply having illustrations of textual material provided more detailed content that enhanced memory [50]. Given that the images used in cigarette warnings were designed to illustrate the health risks, they likely provide memory cues for the warning information. Additional research is needed to determine how to maximize the memorability of health risk information on warnings.

Results of the current research replicate past laboratory findings which have found that the negative affect elicited by graphic warning labels mediates their effect on quit intentions [6, 13, 27]. The current research extends these past investigations by demonstrating that negative affect has significant influences outside the laboratory setting and after many exposures to the warnings. Importantly, results of the current research also provide evidence that images which arouse negative affect may be necessary components of impactful cigarette warning labels where impact is defined as increased risk knowledge, risk perceptions, and quit intentions (see also Wang et al. [51]). In this study, negative affect influenced risk perceptions directly, serving as a simple cue or heuristic. However, negative affect also caused people to more carefully scrutinize smoking-risk information and led to greater perceived label credibility, risk perceptions, and quit intentions. This careful assessment of available information is inconsistent with the labels merely “…browbeat[ing] consumers into quitting” [3]. The impact of negative affect suggests that merely making text-only warnings more salient or including images which do not evoke negative affect may be insufficient to increase smokers’ risk perceptions and quit intentions.

Although the tobacco companies assert that “Americans…are well aware of the health risks of smoking” [3], providing risk information in plain text is less effective than text plus images. Even after exposure to the newly mandated text warnings, participants in this study were able to identify only 3–4 smoking-related risks on average (Table 3). The 2014 Surgeon General’s Report states that smoking harms nearly every organ in the body and identifies at least 35 discrete negative health outcomes caused by smoking [52]. Exposure to warning labels which included graphic images, however, led to greater label memory which was associated, in turn, with increased knowledge of smoking-related risks both at the study’s end and one month later. These findings demonstrate that graphic warning labels can be effective in increasing knowledge about smoking’s dangers, perhaps consistent with research showing the superiority of information accompanied by illustration for enhancing recall of that information [50].

The effects of our warning-label manipulations on smoking risk knowledge, risk perceptions, and quit intentions were indirect and without a significant unmediated effect. Our trial was powered on the expectation of a larger warning label effects on quit intentions than we observed. Additionally, our final sample of 244 analyzed participants falls slightly below our recruitment goal. Nevertheless, the mediated analyses suggest that with a larger sample, experimental effects on quit intentions may have emerged alongside the observed direct effects on affect, label scrutiny, and label credibility. In addition, our finding of a direct effect on label memory suggests that risk knowledge may also be enhanced. It remains for future research with larger samples to demonstrate this conclusively.

Indirect-only effects like those observed in this trial are also important because they suggest the possibility of missing moderators [53]. For example, in the current research, reactance to label content is one possible missing moderator [54]. Such reactance could be the cause of the reduced credibility elicited by the elaborated text condition. Although earlier research has shown that elaborated text such as that used in Canada can enhance warning effects [13], this study’s exclusive focus (in the elaborated text condition) on the very next cigarette entailing risk may have been met with disbelief among life-long smokers who began the study with no intentions to quit. The current research suggests that either additional research is needed to identify the best content for the elaborated text, or that the positive effects of such text are limited to studies with a single exposure to any one warning [13, 35]. Alternatively, the smaller text size in the elaborated-text condition may have been problematic as observational research has demonstrated that warning labels with large, salient text are more impactful than those with smaller and less salient text [55].

Some of our design choices also may have reduced our effect sizes. First, the relatively brief study duration (four weeks) may be inadequate to compete with the power of addiction. Longer trials are expensive, but may be necessary. Second, we chose to use the nine images selected by the FDA for placement on US cigarette packages in their 2011 Final Rule [26]. The images in these warnings are less arousing than those featured on cigarette packages in other countries [56]. Single exposure studies and population level research suggest that placing more disturbing images on graphic warning labels would increase their impact on intentions to change behaviors [51, 56]. Thus, the use of more graphic warnings than those used in the present trial may reveal larger effects. Third, we did not have a true control condition. Participants in the text-only condition received novel warnings that were stronger than those currently featured on US cigarette packaging. In particular, text-only participants received the nine, currently unfamiliar, risk statements mandated by the Family Smoking Prevention and Tobacco Control Act, printed in black text on a white background. Current US warnings are smaller and in colors consistent with the rest of the package. Although we believe that the inclusion of a strong comparison condition is a merit of the current research, our results might have appeared stronger if we had instead compared image conditions to a “current warnings” control group.

The current research was designed to investigate how graphic warning labels might influence smokers at the population level. As such, this trial lacked sufficient statistical power to effectively identify the impact of potential moderating variables. Although past experimental research suggests that the effects of graphic warnings do not vary as a function of demographic characteristics such as race/ethnicity, education, and income [47], this result may prove to be different with a period of prolonged exposure. This possibility should be explored in future research. Some additional variables which future research should consider as potential moderators of the effects observed here include the number of cigarettes participants’ smoke daily, nicotine dependence, duration of smoking, living with a smoker, and personal experience with smoking-related illness. Another plausible moderator is baseline quit intentions. For this study, we only recruited participants who indicated that they were either “somewhat unlikely” or “very unlikely” to quit smoking in the next 30 days during the prescreening phone call. It is possible that graphic warnings might be more impactful for smokers planning to quit in the near future.

This study is the first experiment of which we are aware to investigate the processes by which graphic warning labels influence smokers in a naturalistic setting over an extended period of time. Our method of providing relabeled cigarettes ensured that all participants were exposed to experimental warning labels every time they smoked one of their own cigarettes. However, it is possible that providing participants with cigarettes at no cost may have undermined interest in quitting and worked against our hypotheses (see Table 2). Recent investigations suggest that this can be avoided by requiring participants to provide cigarettes for re-labeling [57] or asking participants to apply experimental labels to their own cigarettes [58]. These techniques may reduce one source of error variance, however, while increasing others (e.g., participant lack of funds or compliance). In addition, if free cigarettes provided disincentive to quit, quit intentions at least should have been influenced equally across all conditions. Overall, the presence of the effects we found speaks to the efficacy of graphic warning labels. Additional investigations are needed to determine the tradeoffs of different methods by which to examine the impact of graphic warning labels in naturalistic contexts.

In particular, the current research provides evidence that negative affect is a crucial ingredient in successful graphic warning labels. It is also the first study to demonstrate that graphic warning labels can elicit greater processing of provided smoking risk information rather than influencing smokers exclusively by “browbeating” them into quitting. Policies requiring such labels have the potential to reduce the number of Americans who smoke. The affect induced by graphic warning labels appears to have utility in communicating more and more credible information, useful to promoting risk perceptions and quit intentions among smokers in the US and around the world.

## Supporting Information

S1 Checklist CONSORT Checklist. (DOC)

S1 Fig Basic Text Warnings. Basic text warnings which were affixed to the side of all cigarette packages distributed in the study. These were the only warning labels affixed to the packages of participants in the text-only condition.
(PDF)

S2 Fig Graphic Warning Labels. Graphic warnings labels were taken from the 2011 FDA final rule. Participants in the graphic images plus basic text condition received these image-text pairings.
(PDF)

S3 Fig Elaborated Text Warning Labels. Graphic warning label images taken from the 2011 FDA final rule modified to include elaborated text. Participants in the graphic image plus elaborated text condition received these warning labels.
(PDF)

S4 Fig Model Testing the Predictions of Hypotheses 1 and 2 with Imputed Data. W3 = Week 3; W6 = Week 6. Model fit statistics: χ^2^(58) = 61.93, p = .338, relative χ^2^ = 1.09, RMSEA = .02 [CI90: .00, .04]; CFI = 1.00; TLI = .99). Path coefficients for the measurement models for Risk Perceptions (Risk 1 = 1.00, Risk 2 = .92**, Risk 3 = 1.21**) and Quit Intentions (Contemplation Ladder = 1.00, 30-Day Quit Intentions = .45**, Quit Desire = .42**).
(PDF)

S5 Fig Model Testing the Predictions of Alternative Model 1. W3 = Week 3; W6 = Week 6. Model fit statistics: χ^2^(58) = 98.84, p = .001, relative χ^2^ = 1.70, RMSEA = .05 [CI90: .04, .07]; CFI = .95; TLI = .93, AIC = 8251.73, BIC = 8433.37. Path coefficients for the measurement models for Risk Perceptions (Risk 1 = 1.00, Risk 2 = .87**, Risk 3 = 1.15**) and Quit Intentions (Contemplation Ladder = 1.00, 30-Day Quit Intentions = .45**, Quit Desire = .41**).
(PDF)

S6 Fig Model Testing the Predictions of Alternative Model 2. W3 = Week 3; W6 = Week 6. Model fit statistics: χ^2^(58) = 104.49, p < .001, relative χ^2^ = 1.80, RMSEA = .06 [CI90: .04, .08]; CFI = .94; TLI = .92, AIC = 8251.73, BIC = 8433.37. Path coefficients for the measurement models for Quit Intentions (Contemplation Ladder = 1.00, 30-Day Quit Intentions = .45**, Quit Desire = .42**) and Risk Perceptions (Risk 1 = 1.00, Risk 2 = .86**, Risk 3 = 1.16**).
(PDF)

S7 Fig Models Testing Hypothesis 3 with Imputed Data. Top: Image presence vs. absence on risk knowledge at week 6 with imputed data, χ^2^ (7) = 12.04, p = .099, RMSEA = .05 (CI 90: .00 to .10), CFI = .80, TLI = .75. Bottom: Image presence vs. absence on risk knowledge at 1 month with imputed data, χ^2^ (7) = 6.53, p = .479, RMSEA = .00 (CI 90: .00 to .07), CFI = 1.00, TLI = 1.04.
(PDF)

S1 File Analyzed Data File. (XLS)

S1 Protocol Complete Trial Protocol. (DOCX)

S1 Table Demographics of Included Participants by Experimental Condition at Randomization. (PDF)

S2 Table Demographic Differences Between Participants Randomized to Conditions Who Did vs. Did Not Complete the Trial. (PDF)
