# Supplementary material for: Graphic Warning Labels Elicit Affective and Thoughtful Responses from Smokers: Results of a Randomized Clinical Trial
Source: PLoS One. 2015 Dec 16;10(12):e0142879. doi: 10.1371/journal.pone.0142879 (PMC4684406; doi:10.1371/journal.pone.0142879)
Supplement: S1 Fig — Basic text warnings which were affixed to the side of all cigarette packages distributed in the study. These were the only warning labels affixed to the packages of participants in the text-only condition. (PDF) [file pone.0142879.s002.pdf]

WARNING:
Cigarettes cause cancer
© U.S. HHS

WARNING:
Tobacco smoke can harm your children.
© U.S. HHS

WARNING:
Smoking can kill you.
© U.S. HHS

WARNING:
Cigarettes are addictive
© U.S. HHS

WARNING: Quitting smoking now greatly reduces serious risks to your health.
© U.S. HHS

WARNING:
Cigarettes cause strokes and heart disease.
© U.S. HHS

WARNING:
Smoking during pregnancy can harm your baby.
© U.S. HHS

WARNING: Tobacco smoke causes fatal lung disease in nonsmokers.
© U.S. HHS

WARNING:
Cigarettes cause fatal lung disease.
© U.S. HHS
