# Supplementary material for: Graphic Warning Labels Elicit Affective and Thoughtful Responses from Smokers: Results of a Randomized Clinical Trial
Source: PLoS One. 2015 Dec 16;10(12):e0142879. doi: 10.1371/journal.pone.0142879 (PMC4684406; doi:10.1371/journal.pone.0142879)
Supplement: S2 Fig — Graphic warnings labels were taken from the 2011 FDA final rule. Participants in the graphic images plus basic text condition received these image-text pairings. (PDF) [file pone.0142879.s003.pdf]

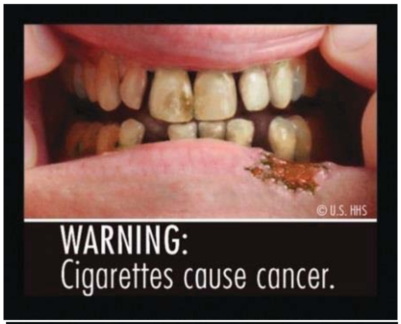
© U.S. HHS
WARNING:
Cigarettes cause cancer.

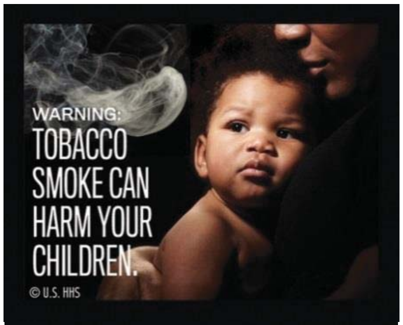
WARNING:
TOBACCO SMOKE CAN HARM YOUR CHILDREN.
© U.S. HHS

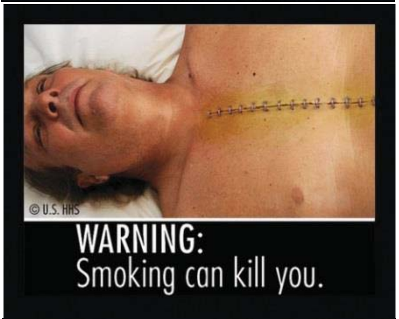
© U.S. HHS
WARNING:
Smoking can kill you.

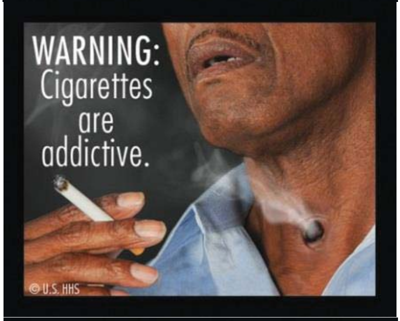
WARNING:
Cigarettes are addictive.
© U.S. HHS

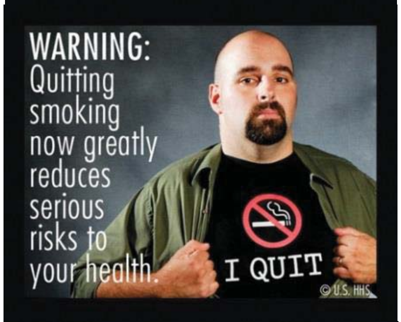
WARNING:
Quitting smoking now greatly reduces serious risks to your health.
I QUIT
© U.S. HHS

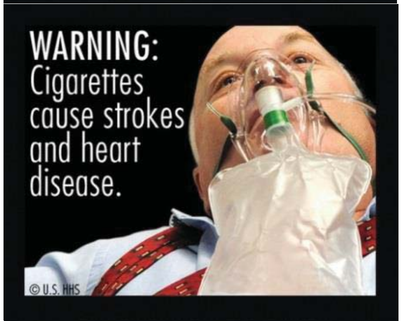
WARNING:
Cigarettes cause strokes and heart disease.
© U.S. HHS

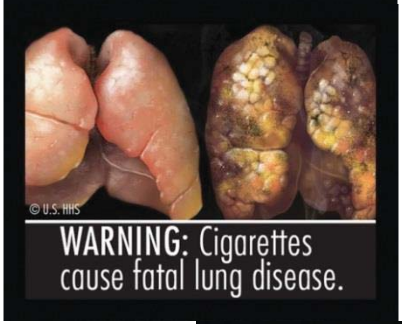
© U.S. HHS
WARNING: Cigarettes cause fatal lung disease.

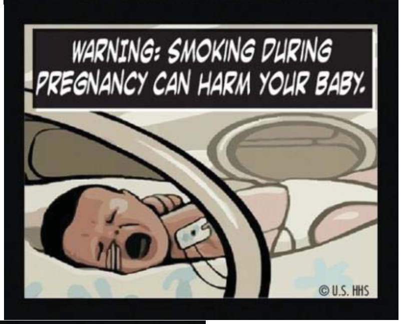
WARNING: SMOKING DURING PREGNANCY CAN HARM YOUR BABY.
© U.S. HHS

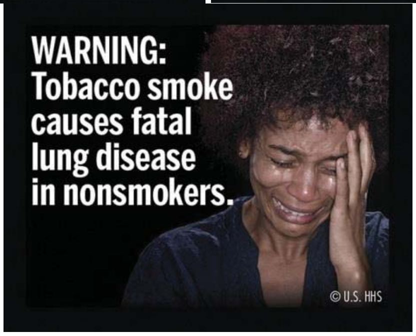
WARNING:
Tobacco smoke causes fatal lung disease in nonsmokers.
© U.S. HHS
