# Supplementary material for: Graphic Warning Labels Elicit Affective and Thoughtful Responses from Smokers: Results of a Randomized Clinical Trial
Source: PLoS One. 2015 Dec 16;10(12):e0142879. doi: 10.1371/journal.pone.0142879 (PMC4684406; doi:10.1371/journal.pone.0142879)
Supplement: S3 Fig — Graphic warning label images taken from the 2011 FDA final rule modified to include elaborated text. Participants in the graphic image plus elaborated text condition received these warning labels. (PDF) [file pone.0142879.s004.pdf]

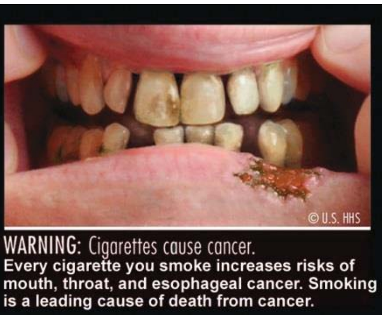
© U.S. HHS
WARNING: Cigarettes cause cancer.
Every cigarette you smoke increases risks of mouth, throat, and esophageal cancer. Smoking is a leading cause of death from cancer.

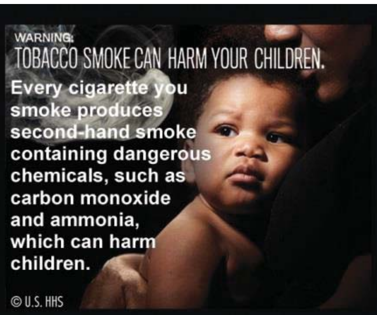
WARNING:
TOBACCO SMOKE CAN HARM YOUR CHILDREN.
Every cigarette you smoke produces second-hand smoke containing dangerous chemicals, such as carbon monoxide and ammonia, which can harm children.
© U.S. HHS

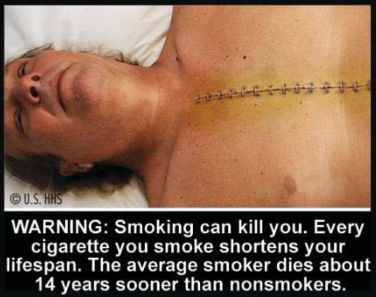
© U.S. HHS
WARNING: Smoking can kill you. Every cigarette you smoke shortens your lifespan. The average smoker dies about 14 years sooner than nonsmokers.

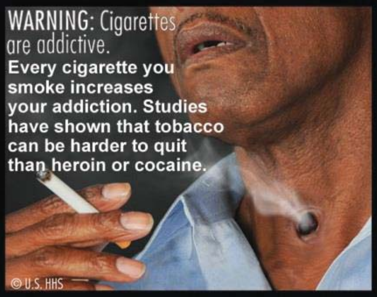
WARNING: Cigarettes are addictive.
Every cigarette you smoke increases your addiction. Studies have shown that tobacco can be harder to quit than heroin or cocaine.
© U.S. HHS

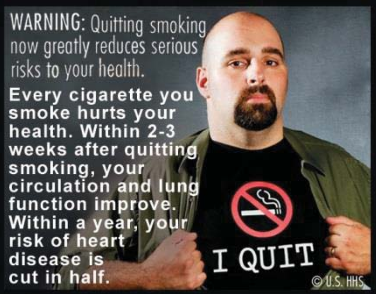
WARNING: Quitting smoking now greatly reduces serious risks to your health.
Every cigarette you smoke hurts your health. Within 2-3 weeks after quitting smoking, your circulation and lung function improve. Within a year, your risk of heart disease is cut in half.
I QUIT
© U.S. HHS

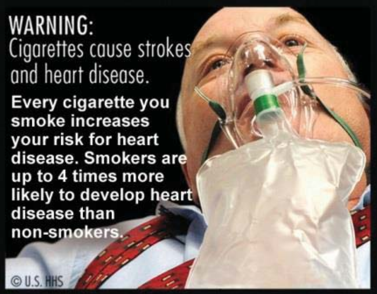
WARNING:
Cigarettes cause strokes and heart disease.
Every cigarette you smoke increases your risk for heart disease. Smokers are up to 4 times more likely to develop heart disease than non-smokers.
© U.S. HHS

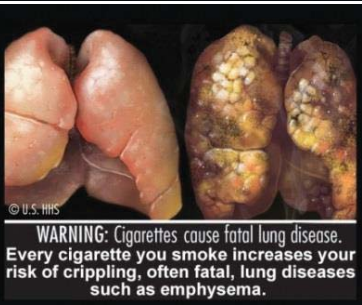
© U.S. HHS
WARNING: Cigarettes cause fatal lung disease.
Every cigarette you smoke increases your risk of crippling, often fatal, lung diseases such as emphysema.

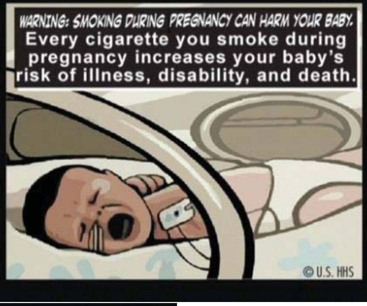
WARNING: SMOKING DURING PREGNANCY CAN HARM YOUR BABY.
Every cigarette you smoke during pregnancy increases your baby's risk of illness, disability, and death.
© U.S. HHS

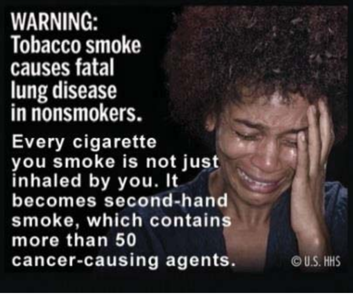
WARNING:
Tobacco smoke causes fatal lung disease in nonsmokers.
Every cigarette you smoke is not just inhaled by you. It becomes second-hand smoke, which contains more than 50 cancer-causing agents.
© U.S. HHS
