# Supplementary figures and images for: Graphic Warning Labels Elicit Affective and Thoughtful Responses from Smokers: Results of a Randomized Clinical Trial
Source: PLoS One. 2015 Dec 16;10(12):e0142879. doi: 10.1371/journal.pone.0142879 (PMC4684406; doi:10.1371/journal.pone.0142879)

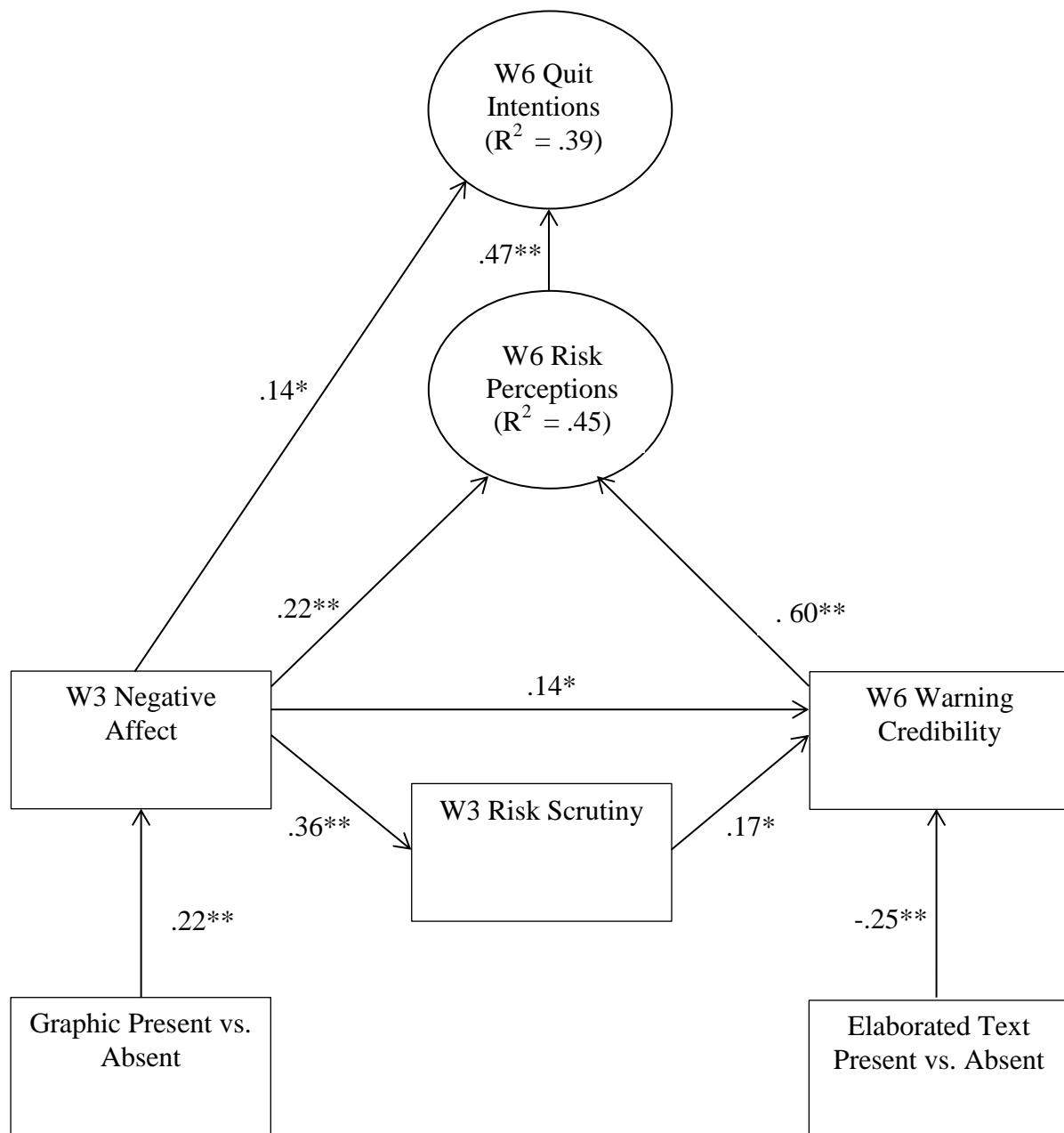

Supplement: S4 Fig — W3 = Week 3; W6 = Week 6. Model fit statistics: χ2(58) = 61.93, p = .338, relative χ2 = 1.09, RMSEA = .02 [CI90: .00, .04]; CFI = 1.00; TLI = .99). Path coefficients for the measurement models for Risk Perceptions (Risk 1 = 1.00, Risk 2 = .92**, Risk 3 = 1.21**) and Quit Intentions (Contemplation Ladder = 1.00, 30-Day Quit Intentions = .45**, Quit Desire = .42**). (PDF) [file pone.0142879.s005.pdf]

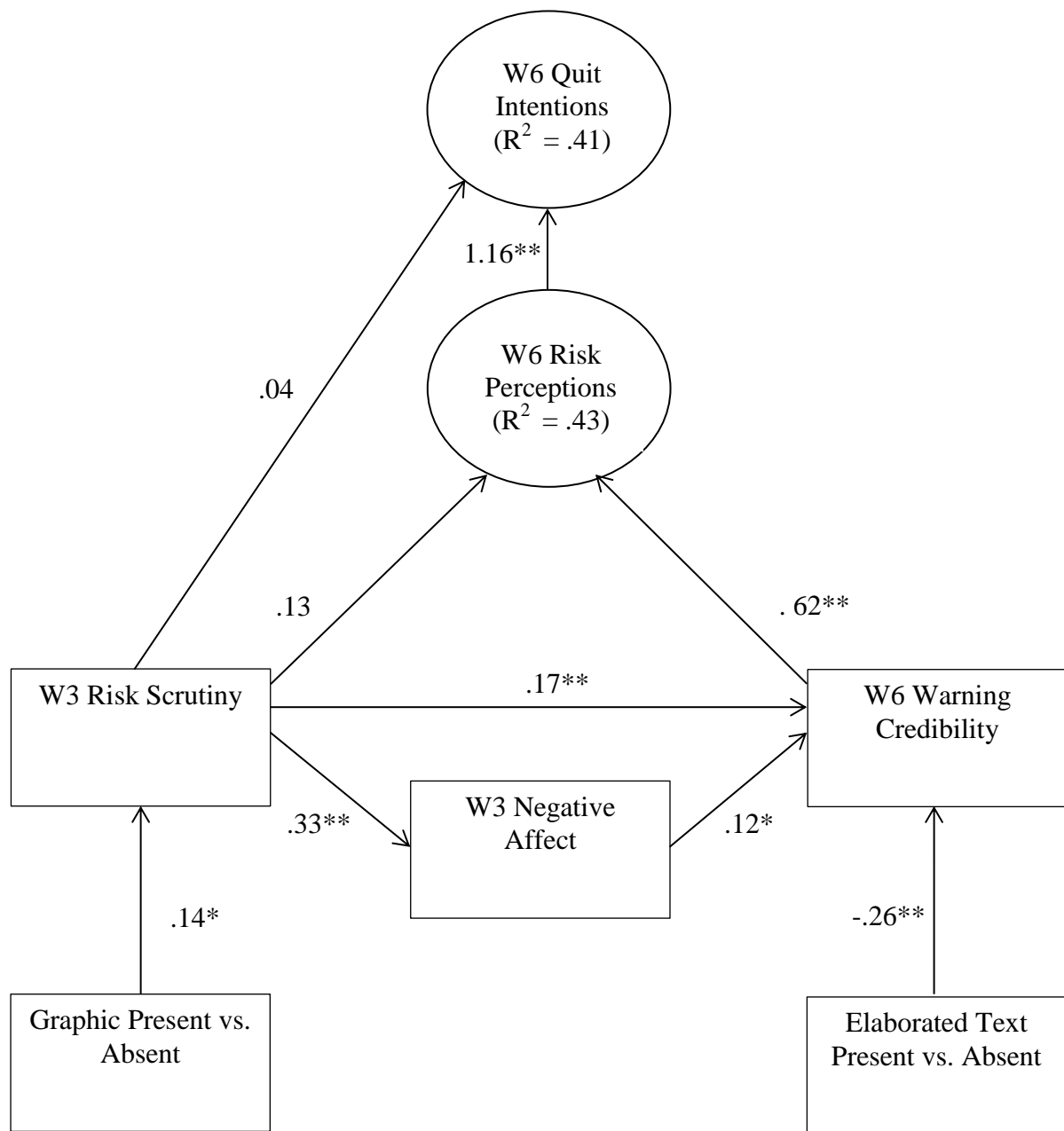

Supplement: S5 Fig — W3 = Week 3; W6 = Week 6. Model fit statistics: χ2(58) = 98.84, p = .001, relative χ2 = 1.70, RMSEA = .05 [CI90: .04, .07]; CFI = .95; TLI = .93, AIC = 8251.73, BIC = 8433.37. Path coefficients for the measurement models for Risk Perceptions (Risk 1 = 1.00, Risk 2 = .87**, Risk 3 = 1.15**) and Quit Intentions (Contemplation Ladder = 1.00, 30-Day Quit Intentions = .45**, Quit Desire = .41**). (PDF) [file pone.0142879.s006.pdf]

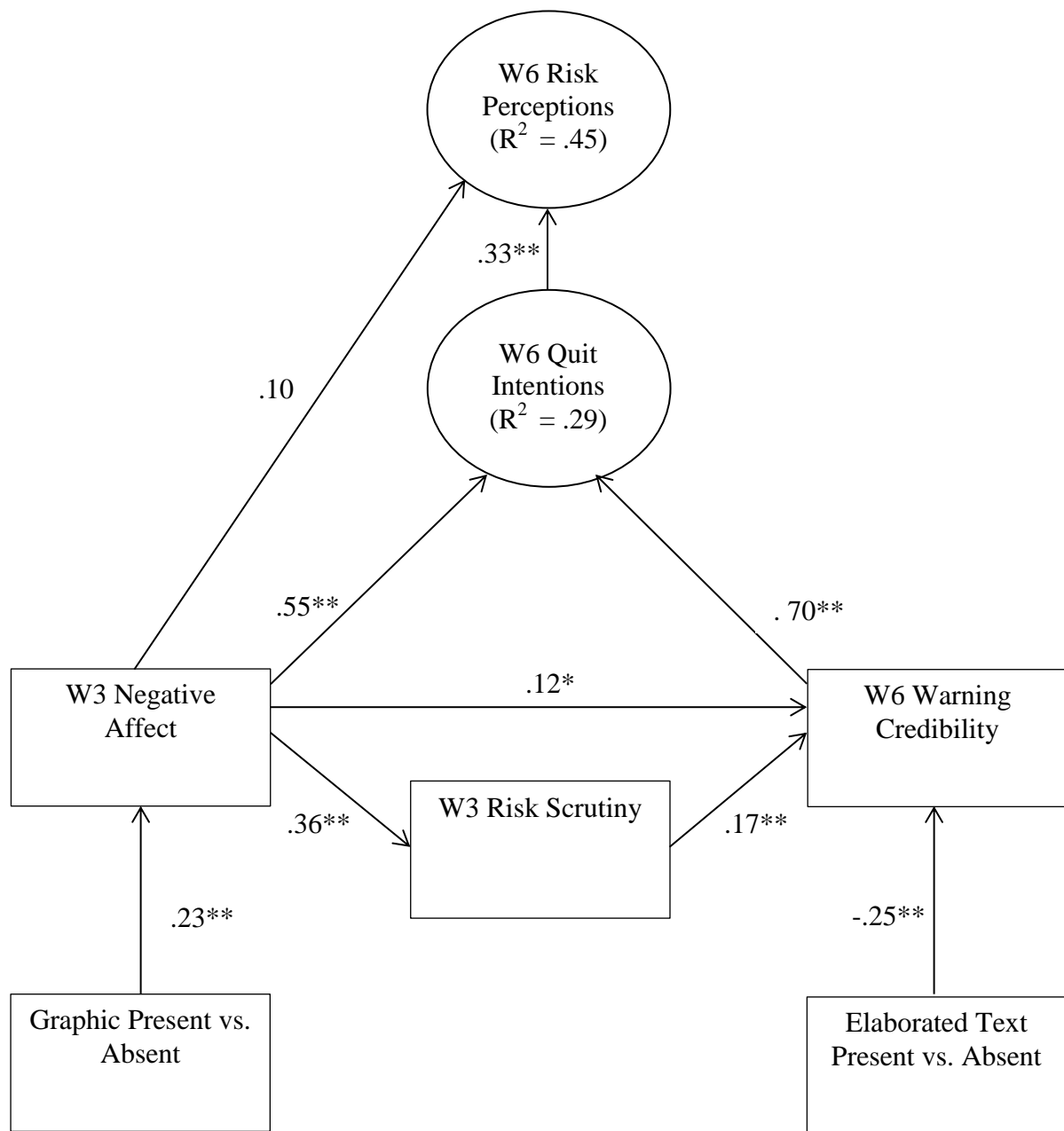

Supplement: S6 Fig — W3 = Week 3; W6 = Week 6. Model fit statistics: χ2(58) = 104.49, p < .001, relative χ2 = 1.80, RMSEA = .06 [CI90: .04, .08]; CFI = .94; TLI = .92, AIC = 8251.73, BIC = 8433.37. Path coefficients for the measurement models for Quit Intentions (Contemplation Ladder = 1.00, 30-Day Quit Intentions = .45**, Quit Desire = .42**) and Risk Perceptions (Risk 1 = 1.00, Risk 2 = .86**, Risk 3 = 1.16**). (PDF) [file pone.0142879.s007.pdf]

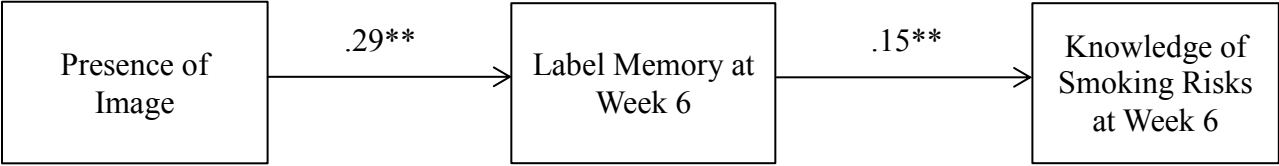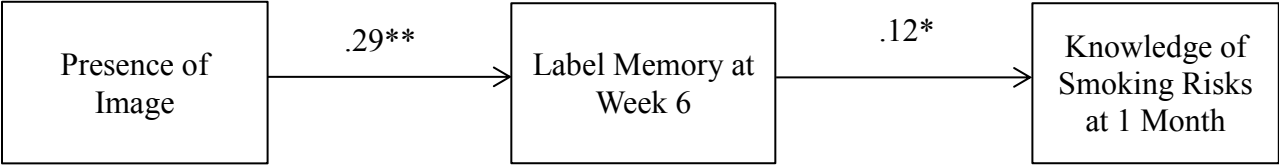

Supplement: S7 Fig — Top: Image presence vs. absence on risk knowledge at week 6 with imputed data, χ2 (7) = 12.04, p = .099, RMSEA = .05 (CI 90: .00 to .10), CFI = .80, TLI = .75. Bottom: Image presence vs. absence on risk knowledge at 1 month with imputed data, χ2 (7) = 6.53, p = .479, RMSEA = .00 (CI 90: .00 to .07), CFI = 1.00, TLI = 1.04. (PDF) [file pone.0142879.s008.pdf]
