# Supplementary material for: Graphic Warning Labels Elicit Affective and Thoughtful Responses from Smokers: Results of a Randomized Clinical Trial
Source: PLoS One. 2015 Dec 16;10(12):e0142879. doi: 10.1371/journal.pone.0142879 (PMC4684406; doi:10.1371/journal.pone.0142879)
Supplement: S1 Protocol — (DOCX) [file pone.0142879.s010.docx]

Graphic Warning Labels Elicit Affective and Thoughtful Responses from Smokers:

Results of a Randomized Clinical Trial

Abigail T. Evans^1*^, Ellen Peters^1^, Andrew A. Strasser^2^, Lydia F. Emery^3^, Kaitlin M. Sheerin^4^, Daniel Romer^5^

^1^Department of Psychology, Ohio State University, Columbus, Ohio, United States

^2^Perelman School of Medicine, University of Pennsylvania, Philadelphia, Pennsylvania, United States

^3^Department of Psychology, Northwestern University, Evanston, Illinois, United States

^4^Department of Psychological Sciences, University of Missouri, Columbia, Missouri, United States

^5^Annenberg School for Communication, University of Pennsylvania, Philadelphia, Pennsylvania, United States

*Corresponding Author

E-mail: [Evans.1339@osu.edu](mailto:Evans.1339@osu.edu) (AE)

Research reported in this publication was supported by grants number P50CA180908 and R01CA157824 from the National Cancer Institute and FDA Center for Tobacco Products (CTP). The content is solely the responsibility of the authors and does not necessarily represent the official views of the NIH or the Food and Drug Administration

1. **Objective**

The introduction of larger and more graphic warnings on cigarette packaging has the potential to significantly reduce smoking rates in the United States, where smokers have not previously been exposed to such warnings. However, the processes by which these warnings influence smokers remain unclear. Thus, the current research was undertaken to investigate the processes that underlie the impact of exposure to different types of graphic warning labels on smokers’ quit intentions in a naturalistic setting and over a relatively long time period.

**Primary Outcome.**

***Quit Intentions.*** The primary outcome of interest for this clinical trial was the impact of graphic warning labels on smokers’ quit intentions. In addition to exploring direct effects of health warning content on quit intentions, the current research explored the processes by which graphic warnings influence quit intentions.

**Secondary Outcomes.**

***Negative Affect.*** The impact of warning labels on feelings about smoking was measured by self-report in weekly laboratory sessions.

***Risk Scrutiny.*** Participants were asked about the extent to which they noticed and paid attention to warning information during each weekly laboratory session.

***Warning Credibility.*** Participants were asked to rate the extent to which they perceived the content of each warning as truthful after two weeks of exposure and at the conclusion of the trial.

***Smoking Behavior.*** Smoking behavior was monitored by requiring participants to return used cigarette filters at each laboratory session and measuring expired breath carbon monoxide.

***Affective Responses to Smoking.*** Ecological momentary assessment was used to track changes in affective responses to smoking outside of the laboratory setting throughout the trial.

***Reactions to Smoking Cues.*** Affective responses to image and text-based smoking cues were measured after two and four weeks of exposure to experimental warning labels.

***Label Memory.*** Participants were asked to record any information they could remember from the warning labels at the conclusion of the trial.

***Smoking Risk Knowledge.*** Smoking risk knowledge was assessed via self-report in an open response task before participants are exposed to the warnings, after two weeks of warning exposure, and at the conclusion of the study.

***Risk Perceptions.*** Feelings of vulnerability to smoking-related risks were assessed by self-report during laboratory sessions.

***Quitline Utilization.*** All participants were provided with a number to call for cessation help. This number was featured on some cigarette packages. Use of this number was monitored by research assistants.

1. **Research Background (adapted from R01CA157824)**

Smoking remains the largest preventable source of mortality in the United States [1]. Although decreasing cigarette use will likely require a range of strategies, one particularly effective way of reaching smokers and potentially decreasing the appeal of cigarettes is through graphic warning labels on cigarette packaging, such as currently used in Canada, Europe, and other parts of the world (see example in Figure 1 below). Because such warnings are delivered at the time of smoking, all smokers are exposed to them. Indeed, pack-a-day smokers are potentially exposed to them more than 7,000 times each year. As required by the Family Smoking Prevention and Tobacco Control Act (Henceforth referred to as, “The Act”), Food and Drug Administration (FDA) issued a “final rule” in June 2011 to impose graphic warning labels that cover 50% of the front and back of all cigarette packs, with the rollout of the program set to occur by October 2012 [2]. The Act specifies the 9 warning statements (e.g., “cigarettes cause cancer”) that must be rotated over time. The FDA also included the national quitline on the warnings. However, going forward, the FDA would have authority to modify the images and any additional text to promote public health. Research on the effectiveness of alternative warning labels thus can have an important influence on FDA practices.


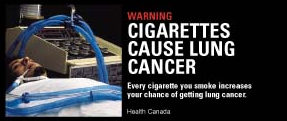


Figure 1. Canadian graphic warning label.

It is unclear what warning-label components would maximize effectiveness in the U.S. Prior field studies examining sets of labels encountered by smokers in non-U.S. countries have shown that responses to warning labels prospectively predicted increases in quit attempts [3,4]. Survey studies have concluded that warning labels are effective [5-8]. Although graphic warning labels are avoided more often than their text-only counter-parts, their avoidance does not seem to undermine quit intentions or attempts [4, 9]. However, no studies appear to have shown that graphic warning labels increase quit attempts or contemplation above levels existing prior to their introduction. The one exception is the finding that attaching a quitline number to the warning increases calls to the line. However, this may merely reflect increased awareness of the quitline rather than effects on the motivation to quit [10, 11]. Canada’s system of graphic warnings coincides with high levels of quit attempts, but this increase in quit attempts has not been shown prospectively or after controlling for other influences [3, 4]. All of the evidence for Canada’s warnings involves responses to the warnings after their introduction.

Initial research conducted by the FDA to test candidate warning images found little evidence of effects of graphic labels on smokers’ immediate quit intentions [12]. In their tests of 36 different images and associated warning statements for two age groups of smokers, only 6 were observed to influence quit intentions and some of these could have been Type 1 errors. Nevertheless, follow-up research by the Annenberg Public Policy Center (APPC) team [13] revealed that these graphic warnings did successfully reduce the **motivation to smoke** as assessed by “wanting a cigarette right now” and “feeling bad about smoking right now.” However, this motivation is only part of what produces behavior change. This problem is anticipated in Protection Motivation Theory (PMT [14]), which recognizes that it is important to consider the effects of health communications on the behavior one wants to discourage **and** on those one wants to encourage. In the case of an addiction such as to nicotine, reducing the motivation to engage in the addictive behavior is insufficient to overcome the cravings and tendencies to relapse that accompany attempts to quit [15]. As Mark Twain once quipped, “Giving up smoking is easy; I’ve done it hundreds of times.” Smokers become increasingly aware of the barriers to quitting as they try to quit. Furthermore, as they disengage from smoking, their sense of vulnerability to the negative health effects of smoking declines because they are actually smoking less. These effects paradoxically reduce the motivation to quit and encourage continued smoking [16]. Thus, the motivation to quit has to be sufficiently strong to overcome these barriers even if one no longer enjoys smoking. [15, 17, 18] As a result, messages are needed to reduce the motivation to smoke and to enhance the motivation to quit.

In more controlled settings, prior experimental research provides evidence that an affective mechanism likely underlies the effectiveness of graphic warnings. This research has found that brief, artificial exposures to graphic labels produced immediate, negative emotional reactions toward cigarettes, including among U.S. participants [19, 20]. However, one element of the warnings used in Canada that has not been exploited by the FDA in its Final Rule is the use of “elaborative text” to emphasize and explain the implications of the simpler warning statements. In the example above from Canada, the statement that cigarettes cause lung cancer is elaborated with the statement that “Every cigarette you smoke increases your chance of getting lung cancer.” From a communication standpoint, this statement serves two purposes: It enhances the importance of the warning by showing how the smoker’s current behavior increases health risks, and it emphasizes the importance of quitting to reduce further risk.

We tested the potential for elaborative text to enhance FDA-proposed warnings in our previous study [13]. We replicated the procedures used by the FDA using different members of the same Internet survey panel. We restricted our test to smokers ages 18+ (we could not assure access to parents of adolescents for permission to conduct the study with their children). We conducted tests for four of the text statements required by the Act: cigarettes cause cancer, smoking can kill you, cigarettes are addictive, and smoking during pregnancy can harm your baby. Each respondent was exposed to a single warning. Our study included the 4 text statements only (controls), 4 FDA-tested images plus the associated text statement, and 8 warnings that each contained an image, text statement, and elaborative text (see APPC website [13]). The elaborative text came primarily from Canadian warnings. Each of the 16 conditions was randomly assigned to at least 150 smokers and separately for ages 18-24 and 25+ (N > 5000 smokers who had smoked at least 100 cigarettes in their lives and were currently smoking every day or some days). The results of that study found support for the addition of elaborative text on various measures of the desire to quit [21]. In addition, the results indicated that the negative affect attached to smoking by the presence of a graphic image separately enhanced the same measures of desires to quit.

Putting these findings together, we predicted that continued exposure to graphic warnings will attach negative affect toward smoking thereby enhancing quit intentions compared to text-only warnings. We base this prediction on considerable psychological research that suggests that the mere presentation of hazard information is not sufficient to motivate perceptions of risk [22] Risk appears most readily communicated by information that arouses emotional associations with the activity [23-25]. Smoking is no exception [26]. Results from Peters, Romer, et al. [20], for example, indicate that brief exposure to Canadian-style labels produces affective responses that transfer to smoking cues and have the potential to convey the risks associated with the use of the product and to decrease smoking. Emotional associations can be readily accessed from memory by the mere presentation of the relevant stimulus [27]. These associations can then work to reduce attraction to the stimulus and motivate cessation. Indeed, emotional associations to smoking appear to be powerful predictors of smoking behavior and may well be causally implicated in efforts to either start or stop smoking [7, 22, 28].

Warnings appear to influence the motivation to smoke, but our model posits that smoker’s motivation to quit also needs to be increased. Slovic [29, 30] has termed this the problem of the “cumulative risk of smoking,” whereby any single cigarette is perceived as harmless, even if it’s recognized that smoking is sufficiently harmful that one should quit at some point. In research conducted with adolescent smokers, Romer et al. [28] showed that the perceived ease of quitting encourages smoking initiation while it simultaneously supports plans to quit at some point in the future. The problem, as already noted, is that as smokers continue in their habit, their addiction makes it much harder to quit than anticipated. These smokers need messages that emphasize the increased risk that accrues from **each additional cigarette**, such as in the example from Canada in Figure 1. In addition, they need ready access to supports that can assist them in their quit attempts. Our design enabled us to observe the effects of warning components that are designed to enhance quit motivations.

We proposed to test two additions that have not been tested by the FDA for the warning label program: elaborated text emphasizing the hazards of each additional cigarette and telephone quitlines. Although the FDA planned to include the national quitline on its warning labels, it is not clear that this information on its own will maximize helpline calls. That is, informing smokers of the quitline may merely give those with enhanced quit intentions the information they need to use the line. What may be needed therefore to maximize quit attempts is enhanced motivation to quit among smokers. These additions were tested in an RCT in which we randomly assigned smokers to different warning label conditions and observed the use of their favorite brand of cigarettes for one month following assignment to condition. A version of the first of these additions elaborative text has already been tested for its effects on immediate reactions to warnings in the large study described above. We found that the addition of this text increased the effectiveness of the warnings, in part, by reducing the motivation to smoke.

Therefore, we proposed an RCT to test the combined and interacting effects of these two components elaborated text emphasizing quitting and quitlines of cigarette warning labels. In the original protocol, smokers were matched on gender, beginning levels of smoking behavior, and intentions to quit and assigned randomly to one of 5 conditions to examine the two components’ effects on quit intentions and attempts to quit smoking. In particular, we proposed to compare a control condition that only shows the warning statements on the side of the pack with 4 graphic image conditions in a 2 X 2 experimental design with the presence or absence of elaborated text and quitline information as the factors. These conditions would enable us to determine whether a quitline is sufficient to motivate quitting apart from elaborative text or whether both are needed to motivate quitting. In addition, elaborative text that primarily focuses on the risks of continuing to smoke may motivate as much quitting as merely putting the quitline information on the pack. And use of elaborated text in place of quit lines would be less expensive to support than quitlines if its efficacy could be proven (e.g., Ohio has already cut funding for its quitline). Our test was designed to determine which of these conditions is most effective at motivating quitting over a much longer period than has been tested heretofore, with actual assessments of smoking and quitting behavior. However, as noted below, we soon discovered that the quitline information was not used by our participants, and for the sake of completing the trial sooner, we asked our funding agencies for permission to drop the quitline component of the trial. Thus, we proceeded to test the three remaining conditions: basic text on the side of the pack vs. two versions of graphic labels (one with and one without elaborated text).

1. **Research Plan and Methodology.**

The present research was conducted using an open-label randomized clinical trial design with an initial five parallel treatment arms. Participants were randomly assigned to receive control text-only warning labels, graphic warning labels featuring the image and text pairings selected by the U.S. FDA in their 2011 final rule, or graphic images and text pairings selected by the U.S. FDA in their 2011 final rule plus additional elaborated text. In addition to the image and text content of the warning label, the presence or absence of a quitline number (that varied by city) was manipulated for participants in conditions which included graphic images. This resulted in a total of 5 treatment arms; Text-only, Image no quitline, Image with quitline, Image with Elaborated text (no quitline), and Image with Elaborated text and quitline.

1. *Power Analysis.*

We used the program G*Power [31] to calculate power estimates with 80% power and a two-tailed alpha of .05. We assumed that compared to the control condition, the four experimental conditions shown in the table below would deviate from control in the following way: .2, .3, .3, .5. That is, we expected quit intentions to increase from .2 to .5 units on the 4-point quit intention scale. This scale has a standard deviation of about 1.0, so we are expecting the intervention to range from small to moderate. With those assumptions, we needed 290 participants in total across both sites. Despite the robust-ness of the data analytic techniques to missing data, we planned to recruit 320 participants in total (160 per site).

With the reduction in the number of conditions from 5 to 3, we recalculated the sample size needed to achieve an effect size of .35 in quit intention. With this reduced study design, we estimated that we would need 260 participants to identify the specified effect size. We also had a baseline measure of quit intention that could be used as a covariate to reduce within cell noise. We recognized that this power estimate assumed little or no design effect across cities, which we believed was likely given that variation within cities will account for nearly all of the variation. Thus, we treated city differences as fixed effects and included tests for city differences and interactions in all analyses


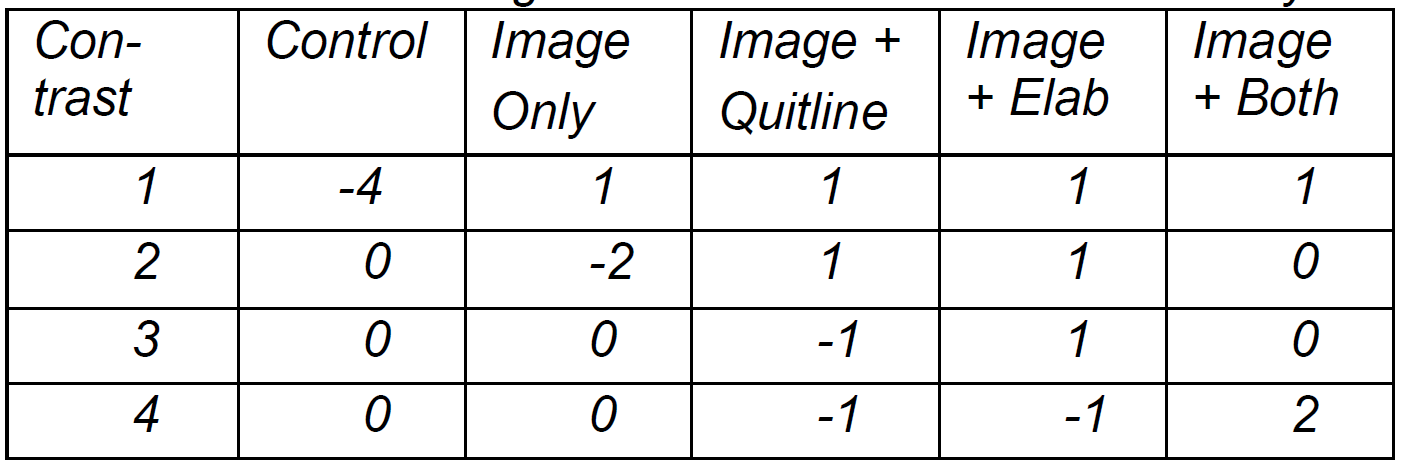


Figure 5. Planned contrasts by experimental condition for power analyses

1. *Recruitment.*

We used IRB-approved recruitment materials (e.g., ads in local newspapers and postings in community sites) to recruit participants. Once scheduled to participate in the experiment, the data were collected over a 35-day time span, with a follow-up call approximately 30 days later. Participants were recruited in two US cities: Columbus, OH and Philadelphia, PA with equal representation from each site.

We used a standardized recruiting script and consent form to recruit the participants. Research assistants were trained in recruitment procedures and had specialized training on how to handle any exceptions. Individuals received an explanation of their rights as research participants, including the right to privacy and their right to withdraw from the study at any time. Participants were assured that their participation in the study was strictly voluntary, and that if they chose to participate, they could change their minds at any time. They were informed about potential benefits and harm.

Participants were asked to sign informed consent forms that explain the project, their rights, how the data would be used in the future, and the plan for how monetary compensation would be provided based on completing the assessments. Participants were told that the cigarettes they are using have warnings that have been approved for use in the United States but that will be put in place at some time in the future. At the conclusion of the study, participants will be told the full purpose of the study.

1. *Inclusion Criteria*

- Smoked between 5 and 40 cigarettes per day and 100 in lifetime
- Between 18 and 65 years of age
- Were not currently trying to quit
- Indicated 1 or 2 when asked “On as scale from 1 to 4 where 1 is ‘very unlikely’ and 4 is ‘very likely,’ how likely do you think it is that you will try to quit smoking within the next 30 days?”

1. *Exclusion Criteria*

- Less than 5ppm expired breath carbon monoxide (CO)
- Smoked 120's or roll-your own or noncommercial cigarettes
- Pregnant or breastfeeding
- History of substance abuse past 5 years
- More than 25 standard alcohol drinks per week
- Use of other nicotine products, such as cigars, nicotine gum
- Current treatment for COPD, cancer, pulmonary disease, alcohol/drug dependence
- Current treatment for a psychiatric condition
- Physical/visual/mental impairment that prevents reading from a computer screen
- Not US resident
- Unable to read English
- English not main language
- Other member of household in study
- Friend who smokes in study
- Unable to attend weekly appointments between 9 am and 6 pm Monday-Friday
- Unable to use hand-held computer to enter daily mood ratings

1. *Intervention*

We created labels that could be pasted on the top 50% of both the front and back of the large (100’s) and regular packs. We also covered the current warning labels on the side of the pack with white labels. In the control condition, we covered one side of the pack with the new warning statements. To accommodate flip-top boxes, we cut the front label to allow the box to be opened. The warnings were downloaded from the FDA’s website and modified to either include our experimental quitline number or to have no quitline number. Based on the requirements of the Act, the nine warning statements were rotated using a Latin square so that each participant would receive approximately the same proportion of each statement across the 28 days of treatment exposure. For the elaborated text conditions, we created messages that reinforced the additional risk that smoking confers with a focus on the disease or condition that is featured in the warning. For example, the Act text message “WARNING: Smoking during pregnancy can harm your baby” will be elaborated by a message that “Continued smoking during pregnancy increases the risk of preterm birth, infant death, and disability.”

1. *Measures*

**Primary Outcome.**

**Quit Intentions.** The primary outcome of this trial was differences between conditions with respect to quit intention. Within the theory of reasoned action [32] as well as PMT, [14] intentions are assessed with a scale that measures the likelihood that the participant will perform a behavior within a specified time period. In the FDA research and our replication study, this was assessed with the question, “How likely do you think it is that you will try to quit smoking within the next 30 days?” with response options on a 4 point scale going from (1) *very unlikely* to (4) *very likely*. The response at the second visit (after smoking their own brand without new warnings) served as a baseline. Responses at the last four visits served as an endpoint of interest. We also measured quit intentions each week using a 9-point contemplation ladder [33] and a measure of non-time-specific desire to quit smoking.

**Secondary Outcomes.**

**Negative Affect.** To identify the processes by which warning labels influence smokers, reactions to warning labels were assessed at each laboratory session after participants began receiving cigarettes in modified packaging. At each visit, participants were asked about the extent to which they liked the warning labels affixed to their pack and the extent to which the packaging affected their feelings about smoking.

**Risk Scrutiny.** At each weekly session, participants were asked about the extent to which they had scrutinized the information on their cigarette warnings. Participants were asked about the extent to which they noticed the warnings, thought about the warnings, and had been affected by the warnings.

**Warning Credibility.** After two and four weeks of exposure to the experimental warning labels, participants viewed each of the warnings which appeared on their cigarette packaging one at a time on a computer monitor. Participants used a 7-point scale to rate the extent to which they perceived the information displayed on each warning as true.

**Smoking Behavior.** To validate the effects of the intervention on actual smoking behavior we measured expired breath carbon monoxide (CO) at the beginning of each visit. This is a commonly used measure of cigarette smoke exposure and has been shown to be sensitive to individual differences in smoking behaviors. [34, 35] CO measures were analyzed in combination with daily cigarette consumption. Expired CO is significantly correlated with carboxyhemoglobin levels [36] and plasma nicotine [37], and is therefore a relatively easier, less expensive proxy than drawing blood. Thus, CO is an important marker of harm exposure [38] that is a practical, often-used measure of smoke exposure in research [37, 38, 39]. Additionally, participants were asked to save the filters of all smoked cigarettes and to return them to the lab at each visit. This provided additional validation of continued tobacco use.

**Affective Responses to Smoking.** One of the methods for assessing the motivation to continue smoking and to quit was experience sampling during the 35 days of the trial. Participants visited the lab on six separate occasions during the study. At the initial visit, each participant was provided with a PDA and received instructions for its use as well as practice. Each week thereafter, research assistants downloaded their experience-sampling data. Participants completed 35 days of computerized experience sampling with three types of interviews: random prompts, smoke events, and non-smoke events. The PDA given to participants was loaded with custom software (ESP [40]) modified by the Ohio State team, allowing the reporting of valence and current mood. To provide a baseline of mood, participants were beeped randomly 5-6 times per day (during waking hours) for a 35-day period and asked about their momentary affective experience (resulting in about 192 measurement moments for baseline mood per participant). At each measurement instance, participants reported their current mood at the time of the beep using 8 emotion-related terms that sample once each of the 8 octants of the valence-arousal space and have been used successfully in previous studies (see Barrett [41]). An additional 3 adjectives (angry, disgusted, regretful) were also included as mean levels of these emotions (similar to happy, sad, and afraid in the original 8 adjectives) that have been related to judgments or decisions in past studies [42] Affect terms were randomized by participant and then presented in fixed order throughout to reduce participant burden. Participants made their ratings on a 5-point rating scale (0=*not at all* to 4=*a great deal*). Participants were told to rate as quickly and accurately as possible the extent to which they feel each emotion at each measurement instance. Response latencies were also recorded. These interviews took about a minute each to complete.

Following Mermelstein et al. [43], participants were also trained to event-record both smoking episodes (even a single puff from a cigarette) and nonsmoking episodes. For a smoking episode, immediately after smoking, participants answered the same questions as before, but in addition they were asked about specific smoking-related items (e.g., how much smoked, feelings about smoking). The mood items were asked twice, once in response to “right now” and once in response to “just before” smoking. A “no-smoke” episode was defined as an occasion when the participant had the opportunity to smoke but made an active decision not to smoke. This assessment allowed us to monitor active attempts to quit and the smoker’s affective reaction to the attempts. Participants answered the same questions as for the smoking episode with the exception of some specific smoking-related items. Using a standardized manual on how to use and respond to the PDA (including how to anticipate/problem solve difficulties), training took about 30 minutes. Mood responses to smoking and no-smoking episodes were compared to baseline mood responses.

Moor, Connelly, and Rogers [44] conducted a PDA usability study with younger (25-30 years) and older adults (75-85 years). They concluded that both age groups could complete tasks such as pressing buttons and identifying and pressing detailed icons with some practice. Older adults preferred larger icons although they could read smaller sizes of even detailed picture icons at 10mm. In the initial visit, we provided a training period in which our participants received training and experience with the method in the presence of an experimenter who could assess comprehension and use of the task on the PDA and provide additional practice as necessary. Based on PDA work by Moor et al. [44] and similar research with touch screen kiosks, we optimized use of the PDAs in our study. Specifically, button sizes on the touch screen were increased in size to a minimum of 10mm and included little detail (e.g. “1” and “2”), font sizes on questions were increased to 18-26 point Arial to maximize readability, and multiple inadvertent touches were treated as single touches with a forced delay. Subjects in previous experience-sampling studies report enjoying and learning from the experience. Methods based on extensive experience reported by Feldman Barrett were used to encourage and maintain such attitudes. Thus, we did not expect any problems with the use of the equipment among the relatively healthy population of smokers we recruited.

**Reactions to Smoking Cues.** Another measure of the motivation to smoke involved the use of the procedure employed by Peters, Romer, et al. [20] to assess affective reactions to smoking cues. When subjects returned to the laboratory the second week to upload their experience-sampling data and obtain cigarettes for the next week, they also completed this task. Mean valence reaction and response times from this second visit (immediately prior to receiving cigarette packages with new warning labels for the first time) were compared to the same measures repeated at the final visit (after four weeks of exposure to the warning labels). We included this assessment to validate measures of mood obtained before and after smoking in the experience-sampling task and to examine affect transfer to smoking cues as the possible underlying mechanism for mood effects. Response time is less subject to self-presentation bias and our task permitted us to assess emotional reactions to a range of smoking cues. The original study found that compared with current U.S. labels, the more graphic Canadian labels produced more negative affective reactions to smoking cues among both smokers and nonsmokers without signs of defensive reactions from smokers. At the second and last visits to the lab, participants were asked to quickly but accurately give their impressions of a series of four smoking images used in Peters, Romer, et al. [20] (e.g., a close-up picture of a burning cigarette in an ashtray) and four smoking-related words (i.e., nicotine, tobacco, cigarette, and smoking). They provided similar reactions to eight food-related images (e.g., meat and vegetables on a plate) and words (e.g. nutrition). For each word and image, participants responded to the question “How does the image or word make you feel?” by pressing one of two buttons for each of four adjective pairs (e.g., good-bad, positive-negative, favorable-unfavorable, and like-dislike). The adjective pairs were presented in random order for each image with a randomized right-left orientation at the bottom of the screen. Response times were recorded from the moment the adjectives appear on the screen to the moment participants pushed one of the two response buttons. Participants who received graphic labels were expected to become more negative to smoking cues. Those who received elaborated text were also expected to exhibit greater negative affect toward smoking.

**Label Memory.** To assess the extent to which graphic images and elaborated text enhance memory for warning information, memory for warning label content was assessed at the conclusion of the trial. Participants were given 9 blank spaces and asked to enter in any information they recalled seeing on their warning labels. Participants were encouraged to list anything they remembered seeing or reading on the labels. This provided a naturalistic measure of free-recall memory.

**Smoking Risk Knowledge.** Smoking risk knowledge was measured before exposure to experimental warning labels, after two weeks of exposure to the warnings, and at the conclusion of the trial. Participants responded to an open ended item which asked them to list as many diseases as they could think of which may be associated with tobacco use. This provided a naturalistic measure of smoking risk knowledge.

**Risk Perceptions.** In addition to listing diseases which may be the result of tobacco use, participants also reported their perceptions of how dangerous smoking is on several Likert-type scales. Participants were asked about the extent to which tobacco use may eventually harm the health of an unspecified person (e.g. “Imagine someone who starts to smoke a pack of cigarettes a day at age 16. How much do you agree with the following statements about this person? There is usually no risk to the person at all for the first few years. 1-7: 1=strongly disagree; 7=strongly agree). Participants were also asked about the extent to which they believe tobacco use puts their own health at risk (“If I continue to smoke, I think my chances of getting a life-threatening illness are:” 1-7: 1=almost zero; 7=almost certain)

**Quitline Utilization.** If participants called the quitline, an answering system requested their ID number or name if the ID is not known. This provided a time-based measure of use of the quitline. It was also set up to forward participants to the appropriate state quitline so that actual assistance for quitting could be received. Since Ohio had ceased to support its quitline at the time of the trial, Columbus residents were transferred to the Pennsylvania site. We predicted that use of the line would be greatest when both elaborative text and the quitline were included in the warning (all participants received the quitline in some form).

**Baseline Measures.** Demographic information (e.g., age, gender, and race), smoking history, nicotine dependence and cigarette brand preference were collected at baseline. Participants also completed measures of health numeracy [45], health literacy (an abbreviated version of the Test of Functional Health Literacy in Adults) [46], decision competence [47], patient activation [48], future time perspective [49, 50] and delay of gratification for risky choices [51]. A single time perspective item, for example, has been significantly linked to policy impact, both cross-sectionally and as a moderator of policy impact, measured pre-post after an enhancement of warnings [51]

In their first and last visits, participants were asked about smoking expectations (Smoking Consequences Questionnaire [52]). The Fagerstrom Test of Nicotine Dependence (FTND) [53] was used to assess nicotine dependence. Greater FTND scores reflect greater nicotine dependence. The FTND is a 6-item self-report measure that has satisfactory internal consistency (α = .64) and high test-retest reliability (*r* = .88) [54]. To assess participant beliefs in their efficacy to quit smoking, we asked both how difficult it would be to quit if they wanted to (as done in our preliminary research) and how effective quitting would be in reducing the risks of disease [20, 21].

1. **References**

1. Centers for Disease Control and Prevention. Tobacco fact sheet. http://www.cdc.gov/tobacco/data_statistics/fact_sheets/fast_facts/index.htm#toll2011.

2. Cigarette health warnings: FDA final rule. http://www.fda.gov/tobaccoproducts/labeling/cigarettewarninglabels/default.htm. Accessed 6/27, 2011.

3. Borland R, Wilson N, Fong GT, et al. Impact of graphic and text warnings on cigarette packs: Findings from four countries over five years. *Tobacco Contro*l. 2009;18(5):358-64.

4. Borland R, Yong HH, Wilson N, et al. How reactions to cigarette packet health warnings influence quitting: Findings from the ITC four-country survey. *Addictio*n. 2009(4):669-75.

5. Fong GT, Hammond D, Hitchman S. The impact of pictures on the effectiveness of tobacco warnings. *Bulletin of the World Health Organizatio*n. 2009;87:640-643.

6. Hammond D, Fong GT, Borland R, Cummings KM, McNeill A, Driezen P. Text and graphic warnings on cigarette packages: Findings from the International Tobacco Control four country study. *American Journal of Preventive Medicin*e. 2007;32(3):202-209.

7. Hammond D, Fong GT, McDonald P, Cameron R, Brown K. Impact of the graphic Canadian warning labels on adult smoking behavior. *Tobacco Contro*l. 2003;12(4):391-5.

8. Hammond D, Fong GT, McNeill A, Borland R, Cummings KM. Effectiveness of cigarette warning labels in informing smokers about the risks of smoking: Findings from the International Tobacco Control (ITC) four country survey. *Tobacco Contro*l. 2006; 15(supple 3):iii19-iii25.

9. Hammond D, Fong GT, McDonald PW, Brown KS, Cameron R. Graphic Canadian cigarette warning labels and adverse outcomes: Evidence from Canadian smokers. *Am J Public Healt*h. 2004;94(8):1442-5.

10. Li J, Grigg M. New Zealand: New graphic warnings encourage registrations with the quitline. *Tob Contro*l. 2009;18(1):72.

11. Miller CL, Hill DJ, Quester PG, Hiller JE. Impact on the Australian quitline of new graphic cigarette pack warnings including the quitline number. *Tob Contro*l. 2009;18(3):235-7.

12. Food and Drug Administration. (1) experimental study of graphic cigarette warning labels, final results report, December 2010 - [required warnings for cigarette packages and advertisements; research report] re FDA-2010-N-0568-0006. http://www.regulations.gov/#!documentDetail;D=FDA-2010-N-0568-0008. Accessed 6/27, 2011.

13. FDA study underestimates impact of graphic tobacco warning labels, Annenberg Public Policy Center research shows. http://www.annenbergpublicpolicycenter.org/NewsDetails.aspx?myId=410. Accessed 6/27/11, 2011.

14. Prentice-Dunn S, Rogers RW. Protection motivation theory. In: Gochman DS, ed. *Handbook of Health Behavior Research I: Personal and Social Determinants.* New York, NY: Plenum Press; 1997:113-132.

15. West R. *Theory of Addiction.* Malden, MA: Blackwell Publishing; 2006.

16. Romer D, Jamieson P, Ahem RK. The catch-22 of smoking and quitting. In: Slovic P, ed. *Smoking: Risk, Perception, & Policy.* Thousand Oaks, CA: Sage Publications; 2001:216-228.

17. Baumeister RF, Heatherton TF, Tice DM. *Losing Control: How and Why People Fail at Self-Regulation.* San Diego, CA: Academic Press; 1994.

18. Benowitz N. The nature of nicotine addiction. In: Slovic P, ed. *Smoking: Risk, Perception, & Policy.* Thousand Oaks, CA: Sage Publications; 2001:159-187.

19. Kees J, Burton S, Andrews C, Kozup J. Understanding how graphic visual warnings work on cigarette packaging. 2010.

20. Peters E, Romer E, Slovic P, et al. The impact and acceptability of Canadian-style cigarette warning labels among U.S. smokers and nonsmokers. *Nicotine Tob Re*s. 2007;9(4):473-81.

21. Emery LF, Romer D, Sheerin KM, Jamieson KH, Peters E. Affective and cognitive mediators of the impact of cigarette warning labels. Nicotine Tob. Res. 2014; 16(3): 263-269.

22. Slovic P, ed. *Smoking: Risk, Perception, and Policy.* Thousand Oaks, CA: Sage; 2001.

23. Slovic P, Peters E. Risk perception and affect. *Curr Dir Psychol Sc*i. 2006;15(6):322-25.

24. Slovic P, Finucane ML, Peters E, MacGregor DG. The affected heuristic. In: In Gilovich T, Griffin D, Kahneman D, eds. *Heuristics and Biases: The Psychology of Intuitive Judgment.* Cambridge, UK: Cambridge Uni Press; 2002:397-420.

25. Peters E, Hibbard JH, Slovic P, Dieckmann NF. Numeracy skill and the communication, comprehension, and use or risk-benefit information. *Health Af*f. 2007;26(3):741-8.

27. Strahan EJ, White K, Fong GT, Fabrigar LR, Zanna MP, Cameron R. Enhancing the effectiveness of tobacco package warning labels: A social psychological perspective. *Tobacco Contro*l. 2002;11(3):183-190.

27. Zajonc RB. Mere exposure: A gateway to the subliminal. *Curr Dir Psychol Sc*i. 2001;10:224-8.

28. Romer D, Jamieson P. The role of perceived risk in starting and stopping smoking. In: Slovic P, ed. *Smoking: Risk, Perception, and Policy.* Sherman Oaks, CA: Sage; 2001:64-80.

29. Slovic P. What does it mean to know a cumulative risk? adolescents' perceptions of short-term and long-term consequences of smoking. *J Behav Decis Ma*k. 2000;13(2):259-266.

30. Slovic P. Do adolescent smokers know the risks? *Duke Law* J. 1998;47(6):1133-1141.

31. Faul F, Erdfelder E, Lang A, Buchner A. G*Power 3: A flexible statistical power analysis program for the social, behavioral, and biomedical sciences. *Behav Res Method*s. 2007;39(2):175-191.

32. Fishbein M, Ajzen I. *Predicting and Changing Behavior: The Reasoned Action Approach.* New York, NY: Taylor & Francis; 2010.

33. Biener, L., & Abrams, D. B. The Contemplation Ladder: validation of a measure of readiness to consider smoking cessation. Health Psychol. 1991; 10(5): 360.

34. Strasser AA, Pickworth WB, Patterson F, Lerman C. Smoking topography predicts abstinence following treatment with nicotine replacement therapy. *Cancer Epidemiol Biomarkers Pre*v. 2004;13(11):1800-4.

35. Strasser AA, Kaufmann V, Jepson C, et al. Effects of different nicotine replacement therapies on postcessation psychological responses. *Addict Beha*v. 2005;30(1):9-17.

36. Jarvis MJ, Belcher M, Vesey C, Hutchison D. Low cost carbon monoxide monitors in smoking assessment. *Thora*x. 1986;41:886-7.

37. Jarvik ME, Caskey NH, Wirshing WC, Madsen DC, Iwamoto-Schaap PN, Elins JL. Bromocriptine reduces cigarette smoking. *Addictio*n. 2000; 95(8):1173-83.

38. Zevin S, Sanders S, Gourlay SG, Peyton III J, Benowitz NL. Cardiovascular effects of carbon monoxide and cigarette smoking. *J Am Coll Cardio*l. 2001;38(6):1633-8.

39. Zacny JP, Stitzer ML, Brown FJ, Yingling JE, Griffiths RR. Human cigarette smoking: Effects of puff and inhalation parameters on smoke exposure. *J Pharmacol Exp The*r. 1987; 240(2):554-64.

40. Barrett DJ, Barrett LF. ESP, the experience sampling program version 4.0. Boston, MA:2005;4.0.

41. Barrett LF. Feelings or words? understanding the content in self-report rating of experienced emotion. *Journal of Personality and Social Psycholog*y. 2004;87(2):266-81

42. Lerner JS, Keltner D. Fear, anger, and risk. *J Per Soc Psycho*l. 2001;89(1):146-59.

43. Mermelstein R, Hedeker D, Flat B, Shiffman S. Real-time data capture and adolscent cigarette smoking. In: Stone A, Shiffman S, Atienza A, Nebeling L, eds. *Science of Real-Time Data Capture: Self-Reports in Health Research.* New York, NY: Oxford Uni Press; 2007:117-35.

44. Moor KA, Connelly KH, Rogers Y. *A Comparative Study of Elderly, Younger, and Chronically Ill Novice PDA Users.* Bloomington: Indiana University; 2004.

45. Lipkus IM, Samsa G, Rimer BK. General performance on a numeracy scale among highly educated samples. *Med Decis Makin*g. 2001;21(1):37-44.

46. Baker DW, Williams MV, Parker RM, Gazmararian JA, Nurss J. "Development of a brief test to measure functional health literacy." *Patient education and counseling* 38, no. 1 (1999): 33-42.

47. Bruine de Bruin W, Parker AM, Fischhoff B. Individual differences in adult decision-making competence. *Journal of Personality and Social Psycholog*y. 2007; 92(5):938-56.

48. Hibbard JH, Stockard J, Mahoney ER, Tusler M. Development of the patient activation measure (PAM): Conceptualizing and measuring activation in patients and consumers. *Health Serv Re*s. 2004;39(4):1005-26.

49. Hall PA, Fong GT. Temporal self-regulation theory: A model for individual health behavior. *Health Psychology Revie*w. 2007;1(1):6-52.

50. Sansone GC, Fong GT, Hall PA, Hastings G. The influence of time perspective on the impact of warning labels: Findings from the ITC United Kingdom survey presented at the 6th national conference on tobacco or health. Montreal, Canada: 2009.

51. Romer D, Duckworth AL, Sznitman S, Park S. Can adolescents learn self-control? Delay of gratification in the development of control over risk taking. *Prev Sci*. 2010;11(3):319-330. Available from: <http://dx.doi.org/10.1007/s11121-010-0171-8>.

52. Brandon TH, Baker TB. The smoking consequences questionnaire: The subjective expected utility of smoking in college students. *Journal of Consulting and Clinical Psycholog*y. 1991;3(3):484-91.

53. Heatherton TF, Kozlowski LT, Frecker RC, Fagerstrom KO. The Fagerstrom test for nicotine dependence: A revision of the Fragerstrom tolerance questionnaire . *Br J Addic*t. 1991;86(9):1119-27.

54. Pomerleau CS, Carton SM, Lutzke ML, Flessland KA, Pomerleau OF. Reliability of the Fagerstrom tolerance questionnaire and the Fagerstrom test for nicotine dependence *Addict Beha*v. 1994;19(1):33-39.
