# Supplementary material for: Graphic Warning Labels Elicit Affective and Thoughtful Responses from Smokers: Results of a Randomized Clinical Trial
Source: PLoS One. 2015 Dec 16;10(12):e0142879. doi: 10.1371/journal.pone.0142879 (PMC4684406; doi:10.1371/journal.pone.0142879)
Supplement: S1 Table — (PDF) [file pone.0142879.s011.pdf]

Table S1. Demographics of included participants by experimental condition at randomization

	Text-only (N = 88)	Graphic Images (N = 105)	Graphic Images and Elaborated Text (N = 96)	Test Statistic (sig.)
**Age**				
	32.15 (11.52)	33.92 (11.49)	34.88 (11.69)	*F*(2, 286) = 1.31, p = .27
**Gender**				
Male	47	58	54	χ^2 (2, 288) = .17, p = .92
Female	41	46	42	
Other	0	1	0	
**Race**				
White	54	65	59	χ^2 (4, 289) = 2.87, p = .58
Black	26	30	33	
Asian	2	1	0	
American Indian	1	1	0	
More than one	3	7	4	
Other	2	1	0	
**Ethnicity**				
Hispanic	3	3	3	χ^2 (2, 289) = .05, p = .98
Non-Hispanic	85	102	93	
**Education**				
Some high school, no degree	7	7	9	χ^2 (2,287) = 5.05, p = .75
High school degree or GED	23	23	26	
Some college, no degree	33	51	40	
Associate's Degree	10	9	5	
Bachelor's Degree	15	13	13	
Master's Degree or higher	0	1	2	
**Self-report Cigarettes Smoked Daily**				
	16.26 (7.48)	17.56 (8.31)	17.02 (8.13)	*F*(2, 268) = .63, p = .53
**Years of Smoking**				
	15.99 (11.86)	17.28 (12.58)	17.79 (11.99)	*F*(2, 285) = .53, p = .59

**Fagerström Test of Nicotine Dependence**	4.42 (1.75)	4.19 (1.88)	4.49 (1.80)	*F*(2, 286) = .75, p = .47
**Breath Carbon Monoxide (CO)**	16.58 (9.68)	18.97 (12.40)	19.15 (9.37)	*F*(2, 286) = 1.66, p = .19

Note. Due to a low number participants in certain groups, some categories were combined in chi-square tests for demographic differences after random assignment. The participant who indicated their gender as "other" was excluded from the chi-square test for gender. Participants who indicted their race as Asian, American Indian, More than one race, or Other, were combined to form a single "other" category. Finally, participants with a Master's degree or higher were combined with participants who held a Bachelor's degree to form a single high-education category.
