# Supplementary material for: Graphic Warning Labels Elicit Affective and Thoughtful Responses from Smokers: Results of a Randomized Clinical Trial
Source: PLoS One. 2015 Dec 16;10(12):e0142879. doi: 10.1371/journal.pone.0142879 (PMC4684406; doi:10.1371/journal.pone.0142879)
Supplement: S2 Table — (PDF) [file pone.0142879.s012.pdf]

Table S2. Demographic differences between participants randomized to conditions who did vs. did not complete the trial

	Did Not Complete Trial (N = 43)	Completed The Trial (N = 244)	Test Statistic (sig.)
**Age**	**30.16**	**34.33**	***t* = -2.21, *p* = .028**
**Gender**			
Male	25	133	χ^2 = .07, *p* = .798
Female	19	110	
**Race**			
White	31	146	χ^2 = 4.35, *p* = .114
Black	8	81	
Other	5	17	
**Ethnicity**			
Hispanic	1	8	
Non-Hispanic	43	236	
**Education**			
No high school degree	3	20	χ^2 = .48, *p* = .923
High School degree	10	61	
Some college	21	103	
College degree	10	60	
**Number of Cigarettes Smoking daily**	**20.57**	**16.36**	***t* = 3.27, *p* = .001**
**Years of Smoking**	14.16	17.55	*t* = -1.69, *p* = .093
**Fagerström Test of Nicotine Dependence**	4.34	4.36	*t* = -.07, *p* = .947
**Breath Carbon Monoxide (CO)**	16.88	18.52	*t* = -.94, p = .347
